# Protein palmitoylation: an emerging regulator of inflammatory signaling and diseases

**DOI:** 10.3389/fimmu.2025.1652741

**Published:** 2025-09-01

**Authors:** Rong Chen, Xiaohua Tang, Ying Wang, Bo Wang, Fei Mao

**Affiliations:** ^1^ Key Laboratory of Medical Science and Laboratory Medicine of Jiangsu Province, School of Medicine, Jiangsu University, Zhenjiang, Jiangsu, China; ^2^ Department of Laboratory Medicine, the Affiliated People’s Hospital, Jiangsu University, Zhenjiang, Jiangsu, China; ^3^ The People’s Hospital of Danyang, Affiliated Danyang Hospital of Nantong University, Zhenjiang, Jiangsu, China

**Keywords:** protein palmitoylation, posttranslational modifications, palmitoyl acyltransferases, acyl protein thioesterase, inflammation, inflammatory diseases

## Abstract

Protein palmitoylation is a reversible lipid modification in which palmitoyl esters are covalently attached to cysteine residues of proteins. It controls various cellular physiological processes and alters protein stability, conformation, localization, membrane binding, and interaction with other effector proteins. Palmitoylation is catalyzed by a group of zinc finger DHHC-containing proteins (ZDHHCs), while the acyl-protein thioesterase family mediates depalmitoylation. Emerging evidence suggests that palmitoylation is critical for inflammatory signaling pathways, where palmitoylation is particularly important in the membrane localization of inflammation-associated proteins. Notably, dysregulation of palmitoylation has been associated with a variety of inflammatory diseases. Here, we provide an overview of the regulatory mechanisms of palmitoylation, explore the emerging role of palmitoylation in inflammatory signaling pathways, and examine the link between dysregulated palmitoylation and the pathogenesis of inflammatory diseases, including inflammatory bowel disease, autoimmune diseases, metabolic dysfunction-associated steatohepatitis, sepsis, Alzheimer’s disease, Parkinson’s disease, and diabetes. Finally, we discuss some of the challenges and opportunities facing the field. Targeting palmitoylation or its associated enzymes serves as a novel therapeutic approach for the treatment of inflammatory diseases.

## 1 Introduction

Inflammation is an important defense response of the body to harmful stimuli. It plays a key role in mitigating damage and maintaining homeostasis by activating immune clearance, initiating tissue repair, and fading in time (1–3). However, uncontrolled or inefficient elimination of the pro-inflammatory response leads to the development of a variety of inflammatory diseases such as inflammatory bowel disease (IBD), hepatitis, sepsis, and neurodegenerative diseases (4, 5). Inflammatory diseases are currently a significant problem for the medical community and economies, affecting a large number of people globally and placing a considerable burden on patients (6). Despite the rapid development of diagnostic methods and therapeutic options in recent years, there is still no fully effective cure for patients with inflammatory diseases (7). The pathogenesis of most inflammatory diseases is extremely complex and involves the aberrant activation of multiple immune and inflammation-related signaling pathways, such as the toll-like receptor pathway (8). To effectively develop diagnostic and therapeutic strategies for treating inflammatory disorders, exploring their pathogenic processes and molecular mechanisms is essential.

Post-translational modifications of proteins are covalent, enzymatic, or non-enzymatic linkages of specific chemical groups on the side chains of amino acids that confer a variety of physiological functions to proteins. These modifications include ubiquitination, phosphorylation, glycosylation, and lipid modifications (9–11). S-palmitoylation is an important protein lipid modification characterized by the covalent binding of palmitic acid (C16 fatty acid) to the sulfhydryl groups of protein cysteine residues via unstable thioester bonds (12). Unlike other lipid modifications, a key property of palmitoylation is its reversibility; thus, palmitoylation can act as a regulator or switch of protein function, similar to ubiquitination or phosphorylation (13). Palmitoylation increases the hydrophobicity of proteins and plays an important role in regulating protein stability, conformation, transport, membrane binding, and interactions with lipids and other proteins. It continues to influence or control many cellular and physiological processes and signaling, including endocytosis, energy metabolism, cell migration and division, innate immunity, and tissue function (14–19).

In recent years, remarkable progress has been made in the study of palmitoylation in inflammation-related cellular and molecular mechanisms, providing additional insights into the pathogenesis and potential therapeutic targets of inflammatory diseases. It is reported that lipopolysaccharide stimulates a strong pro-inflammatory response in immune cells partly through S-palmitoylation of phosphatidylinositol 4-kinase type 2 beta. A growing body of research suggests that the palmitoylation cycle plays a vital role in inflammatory diseases such as IBD, non-alcoholic steatohepatitis, and Alzheimer’s disease (20–22). In this review, we provide insights into the dynamic regulatory mechanisms of palmitoylation and its associated enzymes, systematically analyze the key regulatory roles of palmitoylation in the inflammatory response, and assess its impact on inflammatory diseases and its potential as a therapeutic target.

## 2 Palmitoylation and its regulatory enzymes

Protein lipidation is a post-translational modification process that mainly includes N-myristoylation, S-prenylation, S-palmitoylation. Unlike the other two lipid modifications, S-palmitoylation achieves modification through unstable thioester bonds, a property that makes it the only reversible modification of protein lipidation (23). S-palmitoylation was first reported by Schmidt and Schlesinger in 1979 in the VSV viral G protein (24). It occurs mainly at cysteine residues at the proximal part of the membrane, whose side-chain sulfur atoms form a thioester bond with palmitic acid (25). In addition, palmitoylation also exists as less common types of modifications such as O-palmitoylation (irreversible binding of palmitate to serine residues) and N-palmitoylation (irreversible binding of palmitate to the N-terminus of proteins) (26). S-palmitoylation enhances the hydrophobicity of proteins and significantly facilitates their interactions with biological membranes, thereby precisely regulating their subcellular localization, signaling pathways, and intercellular communication functions (27–30). Data from the SwissPalm database show that there are more than 4000 palmitoylated proteins, including enzymes, receptors, ion channels, transporter proteins, innate immune effectors, and many other soluble and integral membrane proteins (31–33). On a time scale of seconds to hours, palmitoylated substrate proteins can alternate between palmitoylated and depalmitoylated forms, generally in response to signals. S-palmitoylation is catalyzed by DHHC family palmitoyl acyltransferase (PAT) (34, 35) and removed by acyl protein thioesterase (APT) (36) (**Table 1**).

**Table 1:** Palmitoylation enzyme subcellular localizations, substrate profiles, and disease associations.

Enzyme	Subcellular locations	Substrates	Disease Association
ZDHHC1	ER, Golgi	Glycoprotein m6a, GSDMD, IFITM3, NLRP3, Neurochondrin, p53, p110α, IGF2BP1, Mucolipin 3	Viral infection, colorectal cancer, atherosclerosis, prostate cancer
ZDHHC2	Cell membrane, ER, Golgi	Acylglycerol kinase, AKAP79/150, CD151, CD9, CKAP4, Glycoprotein m6a, GSDME, LCK, B-RAF, C-RAF, Hemagglutinin, R7BP, nsP1, PSD95	Renal cell carcinoma, tuberculosis, psoriasis, Alzheimer’s disease, autism, ovarian cancer, hepatocellular carcinoma, bipolar disorder, inflammatory bowel disease, stomach adenocarcinoma, schizophrenia, nasopharyngeal cancer
ZDHHC3	Golgi	Neurochondrin, Cadm4, ACE2, PD-L1, GSDME, ERGIC3, B7-H4, GluA1, IRHOM2, Tim-3, VMP1, SCAP, Integrin α6β4, IFITM3, PI4KIIα, SLC9A2, NCAM, D2R, JAK1, SNAP25/CSP, STING	Demyelinating diseases, viral infections, colorectal cancer, breast tumors, nonalcoholic fatty hepatitis, recurrent miscarriages, hepatocellular carcinoma, clear cell carcinoma of the kidneys
ZDHHC4	Cell membrane, ER, Golgi	D2R, CD36, CD82, MAVS, GSK3β, TRPV1, NFATC4	Breast cancer, diabetes mellitus, metabolic dysfunction-associated steatohepatitis, glioblastoma, lung adenocarcinoma, CLN1 disease
ZDHHC5	Cell membrane	CD36, RIPK1,GSDMD, NOD1/2, CLOCK, NLRP3, EZH2, GRIP1b, ATG9A, PKCδ, Furin, PC7, FAK, Frizzled-5, Flotillin-1, MLKL, lamin A, Desmoglein-2, Plakophilin-3, Phospholemman, STAT3, NCX1, APT1, SMPDL3B, Protocadherin 7, Gαs, Gαi, Trpm7	Glioma, inflammatory bowel disease, fatty liver disease, glioblastoma, neurological disorders, triple-negative breast cancer, NSCLC, diabetic retinopathy
ZDHHC6	ER	MYD88, PPARγ, FLT3-ITD, CLIMP-63, AEG-1, NRas, Diacylglycerol kinase-ϵ	Colorectal cancer, sepsis, leukemia, hepatocellular carcinoma, atypical hemolytic uremic syndrome
ZDHHC7	Golgi, ER	GSDME, IFITM3, PI4KIIα, STAT3, ATG16L1, Diacylglycerol kinase-ϵ, NLRP3, GSDMD, MAVS, CD36, SCRIB, Septin 8, Sprouty 4, APP, Barttin, JAK1, SNAP25/CSP, Fas, Sex steroid receptors, JAM-C, Caveolin-1	Inflammatory bowel disease, infection, prostate cancer, Alzheimer’s disease, clear cell carcinoma of the ovary, non-alcoholic fatty hepatitis, osteosarcoma, chronic stress, hypertension, lung cancer
ZDHHC8	Golgi	Hemagglutinin, D2R, NFATC4, GRIP1b, AnkG-190, GPX4, PSD95, SLC7A11, PICK1	CLN1 disease, autoimmune disease, schizophrenia, cancer, glioma, epilepsy, high-grade plasma ovarian cancer
ZDHHC9	ER, Golgi	GSDMD, GRP78, Rab3gap1, cGAS, GLUT1, CD38, PKG1, PD-L1, NRAS, Ras, β2AR, PIKfyve, β-catenin, STING	Bladder cancer, heart failure, pancreatic cancer, colon cancer, Glioblastoma, triple negative breast cancer, leukemia, colorectal Cancer
ZDHHC11	ER	Neurochondrin, GSDME, ATG2A	Viral infections, fatty liver, Burkitt lymphoma, colorectal cancer, glioma, B-Cell Lymphoma
ZDHHC12	ER, Golgi	NLRP3, claudin-3, MAVS, Gephyrin	Lung adenocarcinoma, ovarian cancer, glioma, systemic lupus erythematosus, osteoporosis
ZDHHC13	Cytoplasmic vesicle, ER, Golgi	SNAP25/CSP, MC1R, ULK1, Drp1, PKM2, MT1-MMP, Huntingtin, EGFR	Melanoma, Parkinson’s disease, cardiovascular disease, atopic dermatitis, osteoporosis, skin cancer, Huntington’s disease, oral squamous cell carcinoma
ZDHHC14	ER, Golgi	PSD93, SLC31A1, β2AR	Viral infection, sclerosing stomach cancer, emphysema, pre-eclampsia
ZDHHC15	Golgi	GSDME, Hemagglutinin, IFITM3, STING, KIBRA, PSD-95, Glycoprotein 130, Neuropilin-2	Autism, glioma, c-MET, glioblastoma
ZDHHC16	ER	DGKϵ, PCSK9, CREB, SETD2	Liver cancer, stroke, glioblastoma multiforme
ZDHHC17	Golgi	GSDME, DGKϵ, SNAP25/CSP, Huntingtin, AKT, HSP90α, Oct4, Smad7, Sprouty-2, SPRED, NLRP3, TrpM7, NMNAT2, Caspase-6	Colorectal cancer, Huntington disease, Non-alcoholic steatohepatitis, Hepatocellular carcinoma, Polycystic ovary syndrome (PCOS), Glioblastoma multiforme, Viral infection, Coronary atherosclerosis, Atypical hemolytic uremic syndrome (AHUS), Type 1 diabetes mellitus, neurodegenerative diseases
ZDHHC18	Golgi	β2AR, cGAS, HRAS, MDH2, VAMP7	Lung adenocarcinoma, B-cell lymphoma, viral infection, renal fibrosis, ovarian cancer, clear cell renal cell carcinoma
ZDHHC19	Cell membrane, Golgi	nsP1, p62, STAT3, SQSTM1, Smad3, Flotillin-1, R-Ras	Lung squamous cell carcinoma, osteosarcoma, sepsis, glioma, cervical cancer
ZDHHC20	Cell membrane, ER, Golgi	Hemagglutinin, EGFR, IFITM3, FASN, YTHDF3, CD80, GPX4, ORAI1, Caveolin-2, MBLAC2	Pancreatic Cancer, Liver Cancer, Inflammation, Viral Infections
ZDHHC21	Cell membrane, Golgi	Sex steroid receptors, Caveolin-1, Adenylate Kinase 2, TRPV2, PIKfyve, α1AR, 5-HT1AR, PLCβ1	Acute myeloid leukemia, diffuse large B-cell lymphoma, Alzheimer’s disease, prion disease, septic injury, and endothelial dysfunction
ZDHHC22	ER, Golgi	mTOR, CCN3	Viral infections, breast cancer, glioma, Alzheimer’s disease
ZDHHC23	Golgi	APT1, PHF2, GFAP, T-bet	Hepatocellular carcinoma, glioma, and neuroinflammatory diseases
ZDHHC24	–	AKT, MAVS	Lung adenocarcinoma, non-alcoholic steatohepatitis, hepatocellular carcinoma
Depalmitoylation			
APT1(LYPA1)	Cytoplasm, cell membrane, nuclear membrane, mitochondria	TRPV1, NCX1, H-Ras, ATG2A, β2AR, p62, Flotillin-1, Caveolin-2, β-catenin, PSD-95, Scamp1, CD36, H-Ras, SQSTM1, BMPR1a	Renal fibrosis, type 2 diabetes mellitus, atherosclerosis, neuroinflammation, and senile osteoporosis
APT2(LYPA2)	Cytoplasm	GSDMD, GPX4, AKT, MAVS, TNF-R1,CKAP4	Viral infections, colitis, lung adenocarcinoma
PPT1	Lysosomes, late endosomes	Sprouty 4,TLR9, AEG-1, GPX1, GFAP, Septin 8	Osteosarcoma, lysosomal storage disease, systemic lupus erythematosus, hepatocellular carcinoma, osteosarcoma, neuronal ceroid lipofuscinoses, neurodegenerative Diseases
PPT2	Lysosome	–	Ovarian cancer, clear cell renal cell carcinoma, neuronal waxy lipofuscinosis
ABHD10	Mitochondria	Peroxiredoxin 5	Alcoholic liver disease, myocardial infarction
ABHD17A/B/C	Cytoplasm, Endosome	NOD2, N-Ras, NLRP3	Crohn’s disease

ER, endoplasmic reticulum; GSDMD, gasdermin-D; IFITM3, interferon-induced transmembrane protein 3; NLRP3, NLR family pyrin structural domain receptor 3; IGF2BP1, insulin-like growth factor 2 mRNA-binding protein 1; AKAP79/150, A-kinase anchoring protein 79/150; CKAP4, Cytoskeleton-Associated Protein 4; GSDME, gasdermin-E; LCK, lymphocyte-specific protein tyrosine kinase; R7BP, regulator of G protein signaling 7 binding protein; nsP1, non-structural protein 1; PSD95, postsynaptic density protein 95; Cadm4, cell adhesion molecule 4; ACE2, angiotensin-converting enzyme 2; ERGIC3, endoplasmic reticulum-golgi intermediate compartment protein 3; GluA1, glutamate ionotropic receptor AMPA type subunit 1; D2R, Dopamine Receptor D2; IRHOM2, inactive rhomboid protein 2; Tim3, T cell immunoglobulin mucin-3; VMP1, vacuole membrane protein 1; SCAP, SREBP cleavage-activating protein; PI4KIIα, phosphatidylinositol 4-kinase; SLC9A2, Solute Carrier Family 9 Member A2, NCAM, neural cell adhesion molecules; MAVS, mitochondrial antiviral-signaling protein; GSK3β, Glycogen synthase kinase 3 beta; TRPV1, transient receptor potential vanilloid 1; NFAT, nuclear factor of activated T-cells; RIPK1, receptor-interacting serine/threonine protein kinase 1; NLRP3, NLR Family Pyrin Domain-Containing 3; CLOCK, Circadian Locomotor Output Cycles Kaput; PKC**δ**, Protein Kinase C Delta; PC7, Proprotein Convertase 7; FAK, Focal Adhesion Kinase; MLKL, mixed lineage kinase domain-like protein; NCX1, Sodium-Calcium Exchanger 1; SMPDL3b, sphingomyelin phosphodiesterase acid-like 3b; TRPM7, transient receptor potential cation channel member 7; PPARγ, peroxisome proliferator-activated receptor gamma; CLIMP-63, cytoskeleton-linking membrane protein of 63 kDa; ATG16L1, autophagy-related 16-like 1; SCRIB, scribble planar cell polarity protein; APP, amyloid precursor protein; JAK1, janus kinase 1; JAM-C, junctional adhesion molecule C; NFATC4, nuclear factor of activated T-cells 4; GRIP1b, glutamate receptor-interacting protein 1b; GPX4, glutathione peroxidase 4; SLC7A11, solute carrier family 7 member 11; PICK1, protein interacting with C kinase 1; GRP78, glucose-regulated protein 78; cGAS, Cyclic GMP-AMP Synthase; GLUT1, glucose transporter 1; PKG1, Protein Kinase G1; β2AR, beta-2 adrenergic receptor; ATG2A, autophagy-related protein 2 homolog A; MC1R, melanocortin 1 receptor; ULK1, Unc-51 Like autophagy activating kinase 1; Drp1, dynamin-related protein 1; PKM2, pyruvate kinase M2; EGFR, epidermal growth factor receptor; SLC31A1, solute carrier family 31 member 1; STING, stimulator of interferon genes; KIBRA; kidney and brain expressed protein; DGKϵ, diacylglycerol kinase epsilon; PCSK9, proprotein convertase subtilisin/kexin type 9; CREB, cyclic AMP response element-binding protein; NMNAT2, nicotinamide mononucleotide adenylyltransferase 2; MDH2, Malate Dehydrogenase 2; **V**AMP7 - Vesicle-Associated Membrane Protein 7; FASN, fatty acid synthase; YTHDF3, YTH domain-containing family protein 3; MBLAC2, metallo-β-lactamase domain-containing protein 2, PLCβ1, Phospholipase C Beta 1; CCN3, Cellular Communication Network Factor 3; PHF2, plant homeodomain finger protein 2; BMPR1a, bone morphogenic protein receptor 1a; GFAP, glial fibrillary acidic protein.

PAT enzymes, also known as ZDHHC enzymes, contain the DHHC (Aspartate-Histidine-Histidine-Cysteine) catalytic tetrapeptide and zinc finger domains (25). Humans have 23 ZDHHC enzymes, named ZDHHC1 through ZDHHC24, excluding ZDHHC10 (37, 38). ZDHHC enzymes were first found in yeast (39) and are highly conserved in all eukaryotes (40). ZDHHC proteins have 4–6 transmembrane structural domains with different membrane localizations, most of which are located in the Golgi and endoplasmic reticulum, and some in the plasma membrane (ZDHHC5, ZDHHC20, and ZDHHC21) (41, 42). ZDHHC enzymes use palmitoyl coenzyme A as a fatty acyl donor (33). Their catalytic mechanism comprises two main steps: an autopalmitoylation step, where palmitoyl coenzyme A attaches to the DHHC motif of the ZDHHC protein, and a transpalmitoylation step, where the palmitoyl group is transferred from the ZDHHC enzyme to the substrate protein, thereby accomplishing its palmitoylation (43). Palmitoylated proteins may be responsive to more than one ZDHHC enzyme, and a single ZDHHC can have multiple substrates. The regulatory mechanisms of how ZDHHC enzymes select specific substrate proteins for modification and their functional redundancy are not fully understood because shared palmitoylated motifs have not yet been identified (15, 44).

Depalmitoylation modifications are mediated by APT1/2 (LYPLA1/2), palmitoyl protein thioesterases (PPT1/PPT2) and α/β hydrolase structural domain-containing proteins 17 (ABHD17A/B/C) and ABDH10 (45, 46). APT resides predominantly in the cytoplasm and shares the same subcellular compartment with ZDHHC enzymes that catalyze its S-palmitoylation. When triggered by a signal, APT is rapidly self-palmitoylated by the ZDHHC enzyme and then shifts from the soluble state to enrichment at the plasma membrane or the inner mitochondrial membrane to perform depalmitoylation at the correct membrane microregion (36, 47). APT1 is the first characterized cytoplasmic thioesterase that catalyzes the depalmitoylation of the alpha subunit of G proteins and the product of proto-oncogene H-Ras *in vitro* (48). APT1 can enable itself and APT2 to undergo palmitoylation, which maintains the normal operation of the palmitoylation cycle (49). Soluble APT2 is susceptible to proteasomal degradation, whereas its membrane-bound form is immune to proteasomal-mediated degradation, and its stable binding to the cell membrane requires three consecutive steps: electrostatic attraction, insertion of a hydrophobic ring, and S-acylation by the palmitoyltransferases ZDHHC3 or ZDHHC7 (50). PPT1 is predominantly localized to lysosomes and late endosomes and may play a role in depalmitoylation of vesicles and lysosomal degradation of S-palmitoylated proteins (47). ABHD17 enzyme is a functional depalmitoylating enzyme in cells, capable of depalmitoylating N-Ras (51) and PAS-95 (52). ABHD17A is itself S-palmitoylated, which is required for plasma membrane binding and proximity to other potential S-palmitoylated protein substrates (53). As a newly identified mitochondrial acylprotein thiolipase, ABHD10 reduces the antioxidant buffering capacity of mitochondria by mediating depalmitoylation of peroxiredoxin 5 (PRDX5) (54). Palmitoylase and depalmitoylase maintain the palmitoylation cycle of proteins and are indispensable in the regulation of protein function and intracellular signal transduction.

## 3 Emerging roles of palmitoylation in inflammatory signaling pathways

### 3.1 TLR pathway

Toll-like receptors (TLRs) play a crucial role in inflammation and host defense by recognizing pathogen-associated molecular patterns (PAMPs) and damage-associated molecular patterns (DAMPs) (55, 56). Ten TLRs (TLR1–10) are known to exist in humans. The cell membrane contains TLR1-2, TLR4-6, and TLR10. Intracellular endosomes are the anchor for TLR3 and TLR7-9 (8). TLR (excluding TLR3) activation stimulates interleukin-1 receptor-associated kinase (IRAK)1 and IRAK4 by recruiting myeloid differentiation primary response protein 88 (MYD88). IRAK1 and IRAK4 then trigger downstream signaling proteins, such as TAK1-binding protein 1 (TAB1), transforming growth factor-β-activated protein kinase 1(TAK1), tumor necrosis factor receptor-associated factor 6 (TRAF6), and TAB2. As a result, NF-κB and MAP kinases (MAPK) are activated, which causes the synthesis of chemokines, type I interferons, and inflammatory cytokines, ultimately leading to inflammation (57–59).

**Figure 1:**
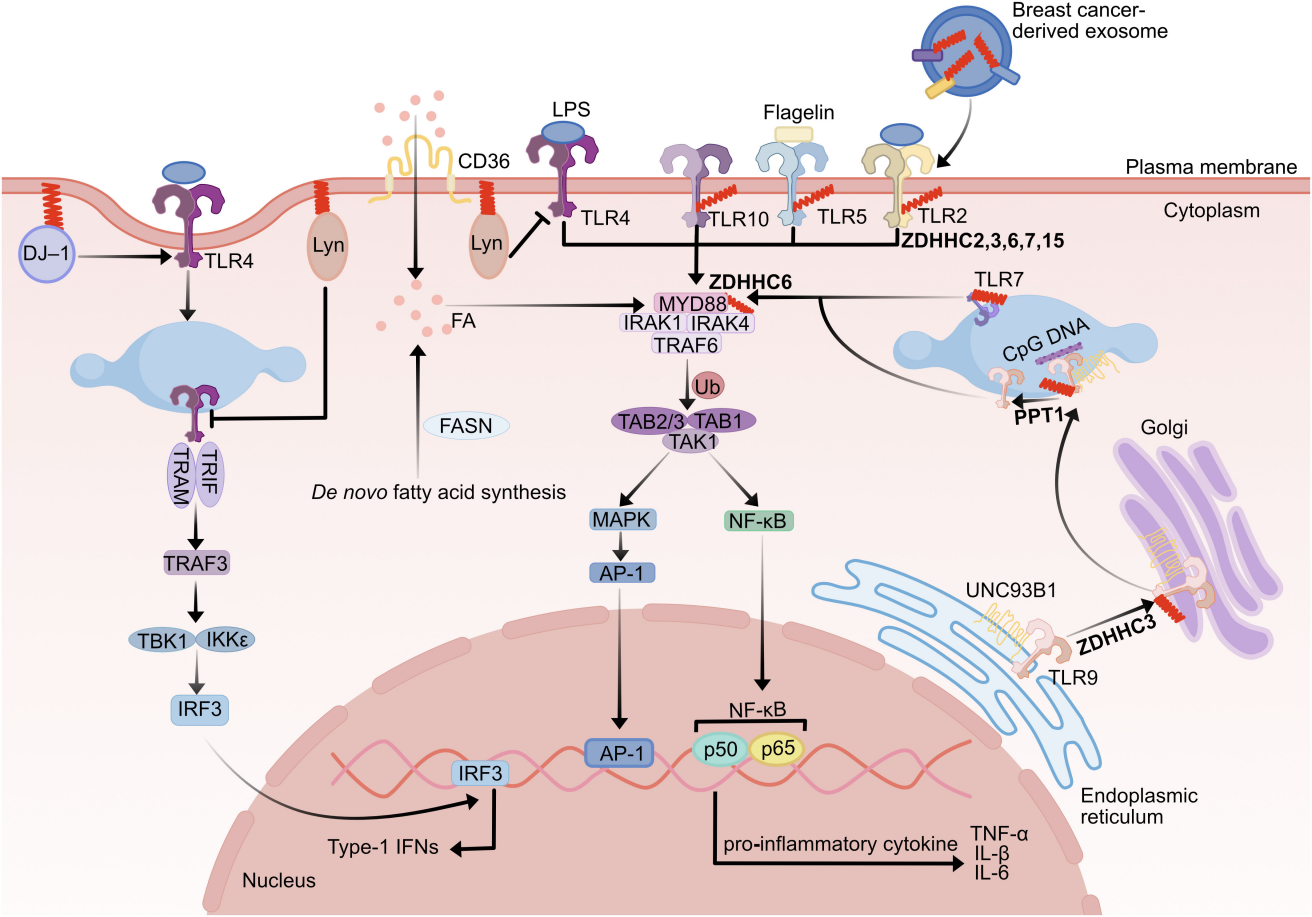
Effect of palmitoylation on the TLR pathway. TLR2, 5, 7, 9, and 10 are palmitoylated, critically regulating their membrane localization, trafficking, and downstream signaling. The palmitoylation of TLR2 by ZDHHC2, 3, 6, 7, and 15 is necessary for its full activation and NF-κB signaling. Palmitoylation of TLR9 by ZDHHC3 in the Golgi facilitates its endosomal trafficking and is followed by depalmitoylation by PPT1, which releases TLR9 from UNC93B1 for signaling. DJ-1 protein palmitoylation promotes DJ-1 translocation to lipid rafts, which contributes to TLR4 endocytosis to inhibit its activation. Palmitoylated proteins on the exosome surface of human breast cancer cells activate the pro-inflammatory signaling pathway of TLR2. Lyn kinase palmitoylation upon LPS stimulation negatively regulates TLR4 signaling. MYD88 palmitoylation enhances IRAK4 recruitment and downstream NF-κB activation, a process supported by both *de novo* fatty acid synthesis and CD36-mediated exogenous fatty acid uptake.TLR, toll-like receptor; ZDHHC, zinc finger DHHC domain-containing protein; PPT1, palmitoyl-protein thioesterase 1, TRAM, trif-related adapter molecule; TRIF, TIR domain-containing adapter inducing IFNβ; TRAF, tumor necrosis factor receptor associated factor; TBK1, TANK-binding kinase 1, IRF3, interferon regulatory factor3; MYD88, myeloid differentiation primary response protein 88; IRAK, interleukin-1 receptor-associated kinase; TAK1, transforming growth factor-β-activated protein kinase 1; TAB1, TAK1-binding protein; MAPK, MAP kinases; AP-1, activating protein-1; IFN, Interferon; NF-κB, nuclear factor κB; LPS, lipopolysaccharide; Ub, ubiquitin; FA, fatty acid.

Palmitoylation is important for TLR recruitment and activation of its downstream pathway (**Table 2**). Palmitoylation modifications of TLR7 and TLR9 can regulate their transport and membrane stability, thus influencing the activation of downstream signaling pathways (60). In particular, there exists a palmitoylation cycle of TLR9 mediated by Golgi-resident ZDHHC3 and lysosome-resident PPT1, which regulates the binding of TLR9 to CpG ligands and cytokine production in plasmacytoid dendritic cells (pDC) and macrophages (60). Membrane localization of TLR2 is also dependent on its palmitoylation modification. Pharmacological inhibition and mutation of the Cys609 site lead to a reduction in the cell membrane sites of TLR2 and impair the transcription of the NF-κB gene as well as the release of pro-inflammatory cytokines, such as TNF-α and IL-6 in dendritic cells and fibroblasts (61). Furthermore, TLR10 and TLR2 form a physical interaction complex that controls TLR2 responses. Additionally, TLR10 has a high palmitoylation signal as compared to its total protein level, indicating that palmitoylation can regulate the TLR2 signaling network’s activity via two different mechanisms (direct modulation of TLR2 function and interaction with TLR10) (61) (**Figure 1**).

**Table 2:** Function and disease associations of palmitoylation in inflammation-related proteins.

Protein	Palmitoylation sites (Human/Mouse)	PATs/APTs	Functional mechanism	Related diseases	References
TLR9	C258, 265 (Mouse)	ZDHHC3/PPT1	Palmitoylation regulates TLR9 trafficking to endosomes;Depalmitoylation facilitates TLR9 release from UNC93B1	SLE	(60)
TLR2	C609 (Human)	ZDHHC2,3,6,7,15	Promotes TLR2 transport to the cell membrane	Infection	(61)
MYD88	C113, 274 (Human)	ZDHHC6	Regulates neutrophil Chemotaxis and promotes IRAK4 recruitment and downstream signaling	Sepsis	(62)
Lyn	C3 (Human/Mouse)	Unknown	Inhibition of LPS-induced TLR4 signaling	Bacterial infection	(63)
GRK6	–	Unknown	Anchors GRK6 to the cell membrane	Liver damage	(64)
DJ-1	C46, 53, 106 (Human)	Unknown	Localization of DJ-1 into lipid rafts	Parkinson’s disease	(65)
NOD1	C558, 567, 592 (human/mouse)	ZDHHC5	Recruitment of NOD1 to bacterial-containing endosomes and other intracellular membranes	–	(66)
NOD2	C395, 1033 (human/mouse)	ZDHHC5	Recruitment of NOD2 to bacterial-containing endosomes and other intracellular membranes; Restriction of NOD2 autophagic degradation mediated by the translocation recognition receptor SQSTM1/p62	Crohn’s disease	(66, 67)
cGAS	C474 (Human)	ZDHHC18	Inhibits cGAS DNA binding and dimerisation	DNA viral infection	(68)
	C404, 405 (Human)	ZDHHC9/LYPLA1	Promotes cGAS dimerization and activation	Tumor	(69)
STING	C88/91 (human,Mouse)	ZDHHC3,7,15	Activates STING-induced type I interferon response at the Golgi	SAVI	(70)
NLRP3	C130/C126 (Human/Mouse)	ZDHHC1,3,5,7/ APT2	Promotes resting-state NLRP3 localization to the TGN and activated-state NLRP3 localization to the dTGN	Endotoxic shock; Peritonitis	(71–73)
	C901/C898 (Human/Mouse)	Unknown	Promotes NLRP3 Translocation to dTGN Vesicles	–	(117)
	C958/C955 (Human/Mouse)	ZDHHC1	Localizes NLRP3 to TGN under resting conditions; Transiently targets NLRP3 to mitochondria in early activation	Endotoxic shock	(73)
	C837, 838/C834, 835 (Human/Mouse)	ZDHHC5/ABHD17A	Promotes NLRP3-NEK7 interaction	Autoinflammatory disease	(74)
	C419/C415 (Human/Mouse)	ZDHHC17	Promotes NLRP3-NEK7 interaction	IBD	(20)
	C130, 261/C126 (Human/Mouse)	ZDHHC7/ABHD13	Enhances NLRP3 phase separation	endotoxic shock	(75)
	C844/C841 (Human/Mouse)	ZDHHC12	Enhances NLRP3 degradation	Endotoxic shock; Peritonitis	(76)
	C8/C6 (Human/Mouse)	PPT1	Enhances NLRP3 stability	Diabetes mellitus	(77)
GSDMD	C191/C192 (Human, Mouse)	ZDHHC5,7,9/APT2	Increases the interaction of GSDMD with caspase-1; Controls membrane translocation and oligomerization of GSDMD-NT	Lethal sepsis; bacterial infection	(78, 79)
GSDME	C407, 408 (Human)	ZDHHC2,7,11,15	Blocks the interaction and promotes the dissociation of GSDME-NT and GSDME-CT	Radiotherapy	(80)

TLR, toll-like receptor; ZDHHC, zinc finger DHHC domain-containing protein; MYD88, myeloid differentiation primary response protein 88; GRK6, G protein-coupled receptor kinase 6; NOD1/2, nucleotide-binding oligomerization domain-containing proteins1/2; cGAS, cyclic GMP-AMP synthase; STING, stimulator of interferon genes; NLRP3, NLR family pyrin structural domain receptor 3; NEK7, NIMA-related kinase 7; TGN, trans-Golgi network; dTGN, dispersed trans-Golgi network; GSDMD, gasdermin-D; GDMD-NT, N-terminal domain; GSDMD-CT, C-terminal domain; GSDME, gasderminE; SLE, systemic lupus erythematosus; IBD, Inflammatory bowel disease; SAVI, STING-associated vasculopathy with onset in infancy.

In addition to the TLR itself, several downstream signals in its signaling pathway are modified by palmitoylation. MYD88 palmitoylation is required for TLR signaling activation and is regulated by fatty acid synthase (FASN)-mediated fatty acid *de novo* synthesis and CD36-mediated exogenous fatty acid incorporation. Knockdown of *ZDHHC6* in macrophages inhibits MYD88 palmitoylation modification and lipopolysaccharide (LPS) responsiveness; therefore, TLR-mediated inflammation can be reduced by inhibiting MYD88 palmitoylation or by limiting endogenous palmitate available for protein modification (62). Although there is no direct evidence for palmitoylation of TRAF6, it has been demonstrated that ZDHHC11 can interact with TRAF6 to promote its oligomerization and ligase activity, followed by activation of the TAK1 and IκB kinase (IKK) complexes, which promotes NF-kB signaling activation in HEK293T cells (81). Meanwhile, palmitoylation can indirectly regulate the TLR receptor signaling pathway’s activation. Lyn palmitoylation negatively regulates LPS-induced TLR4 signaling to block the activity of NF-κB and interferon regulatory factor 3 (IRF3) signaling pathways in RAW264 cells (63). DJ-1 protein also contributes to TLR4 receptor endocytosis by palmitoylating targeted lipid rafts to diminish the intensity of its signaling activation (65). Palmitoylation mediates protein sorting into extracellular vesicles (EVs) (82), and palmitoylated proteins on breast cancer-derived exosomes activate TLR2 signaling to drive NF-κB activation (83). This suggests specific palmitoylated proteins are selectively incorporated into EVs to induce inflammatory responses (83). In Kupffer cells, LPS is able to increase the level of G protein-coupled receptor kinases 6 (GRK6) palmitoylation, promoting its translocation to the cell membrane and inducing an inflammatory response; however, inhibition of GRK6 palmitoylation may impair LPS stimulation of TLR4-mediated inflammatory responses (64).

### 3.2 NOD1/2 pathway

NOD1 and NOD2, two well-characterized pattern recognition receptors (PRRs) in the nucleotide-binding oligomerization domain (NOD)-like receptor (NLR) family, sense bacterial peptidoglycan (PGN) and activate intracellular signaling pathways that drive pro-inflammatory and antibacterial responses (84). Host cells can internalize PGN through a variety of pathways, such as internalizing bacteria through phagocytosis (85). Upon ligand recognition, NOD1/2 recruits receptor-interacting serine/threonine-protein kinase 2 (RIPK2) via CARD-CARD interactions, which further mediates TAK1 recruitment and activation, followed by activation of NF-κB and MAPK signaling, leading to transcriptional up-regulation of pro-inflammatory and host defense genes (86–88). Several studies have shown that disruption of the NOD1/2 signaling pathway is involved in the development of several inflammatory diseases, such as IBD (89).

**Figure 2:**
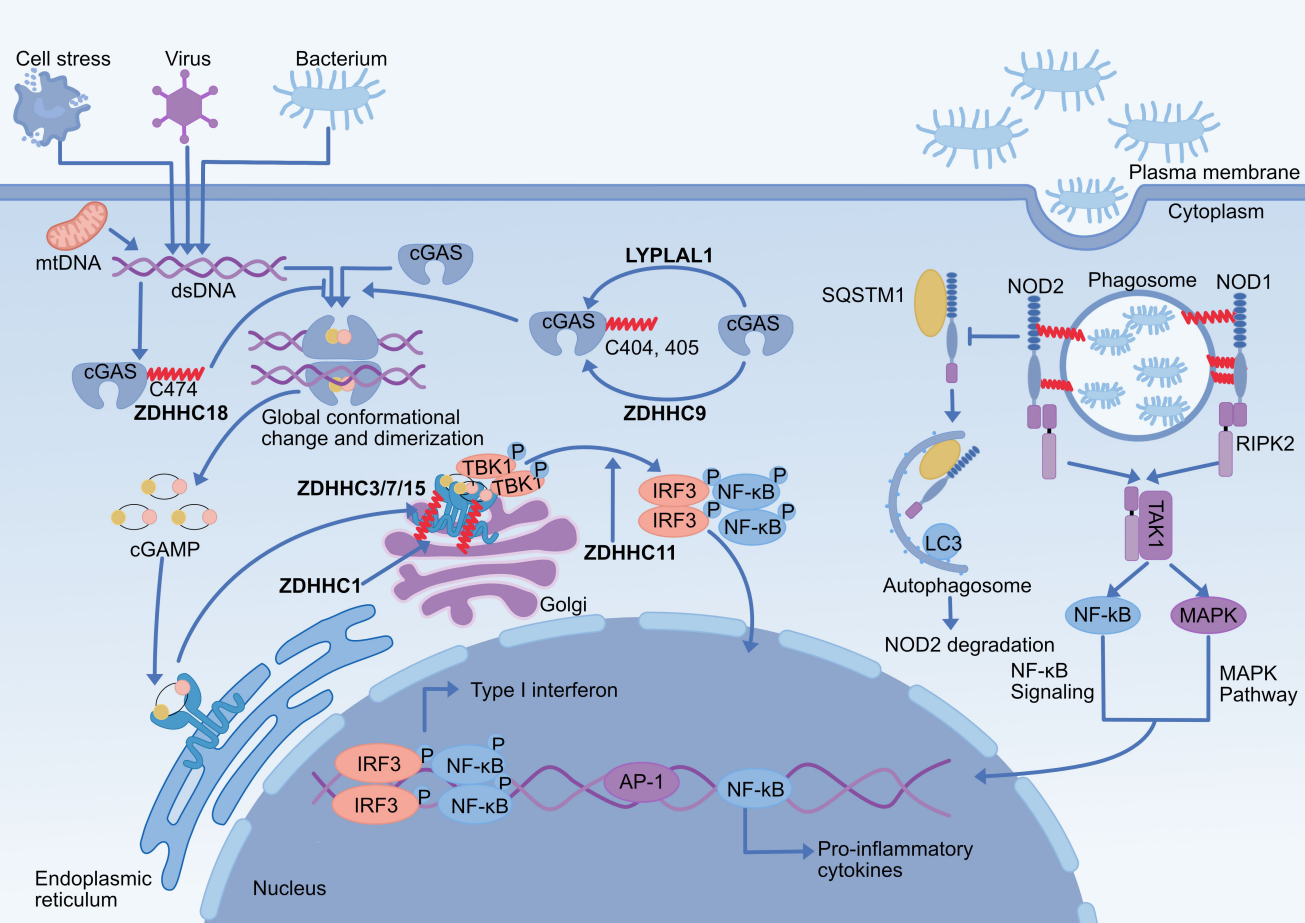
Effect of palmitoylation on NOD1/2 and cGAS-STING pathway. (1) S-palmitoylation of NOD1/2 is critical for their ability to respond to peptidoglycans and to mount an effective immune response. NOD1 (Cys558/567/952) and NOD2 (Cys395/1033) are palmitoylated by zDHHC5 at bacteria-containing endosomes, which promotes their translocation to endosomal membranes and subsequent signal transduction. NOD2 palmitoylation inhibits the SQSTM1/p62-mediated autophagic degradation of NOD2. (2) cGAS Cys474 palmitoylation reduces the interaction of cGAS with double-stranded DNA by limiting its enzymatic activity, further inhibiting cGAS dimerization, and double-stranded DNA promotes palmitoylation modification of cGAS. The cGAS Cys405/405 undergoes ZDHHC9-mediated palmitoylation, promoting its dimerization and activation, and LYPLAL1 mediates depalmitoylation of cGAS. (3) ZDHHC1 promotes STING dimerization and ZDHHC11 regulates recruitment of IRF3 to STING. (4) Palmitoylation of STING Cys 88/91 on the Golgi apparatus contributes to the activation of STING-dependent downstream signaling for the type I interferon response, which is catalyzed by ZDHHC 3/7/15. ZDHHC, zinc finger DHHC domain-containing protein; LYPLAL1, lysophospholipase-like 1; cGAS, cyclic GMP-AMP synthase; STING, stimulator of interferon genes; dsDNA, double-stranded DNA; mtDNA, mitochondrial DNA; cGAMP, cyclic GMP-AMP; TBK1, TANK-binding kinase 1; IRF3, interferon regulatory factor3; NOD1/2, nucleotide-binding oligomerization domain-containing proteins1/2; SQSTM1/p62, sequestosome 1;RIPK2, receptor-interacting serine/threonine-protein kinase 2; TAK1, transforming growth factor-β-activated protein kinase 1.

Membrane localization of NOD1/2 is essential for its mediated immune signaling. Lu et al. demonstrated that membrane localization of NOD1/2 is regulated by palmitoylation (**Table 1**). NOD1/2 undergoes palmitoylation at multiple cysteine residues, facilitating its binding to phagosomal membranes in response to peptidoglycan and triggering an effective immune response. ZDHHC5, a palmitoyltransferase localized in the phagolysosome, is indispensable for the palmitoylation of NOD1/2 (66). Furthermore, palmitoylation of NOD2 by ZDHHC5 limits NOD2 autophagic degradation mediated by the transporter recognition receptor SQSTM1/p62, thereby enhancing NOD2 stability and promoting NOD2-mediated inflammatory responses (67). Existing studies have shown that palmitoylation regulates the NOD1/2 signaling pathway by both promoting NOD1/2 membrane translocation and inhibiting and suppressing NOD2 degradation, which together mediate NOD1/2-dependent host defense responses, providing new ideas for the diagnosis and treatment of inflammatory diseases. (**Figure 2**).

### 3.3 cGAS-STING pathway

The cyclic GMP-AMP synthase (cGAS)- stimulator of interferon genes (STING) signaling pathway can sense and control cells’ capacity to trigger innate immune activation in response to microbial and host-derived DNA (90), and it has become a crucial signal for inflammation in the context of infection, cellular stress, and tissue damage (91). Aberrant cGAS-STING activation leads to excessive and sustained production of type I interferon, which mediates local or systemic inflammation and is thus involved in the development of many inflammatory disease processes (92). In this pathway, cGAS dimerizes upon binding to cytoplasmic double-stranded DNA and is catalyzed to synthesize 2’,3’- cyclic GMP-AMP (cGAMP), which acts as a second messenger to bind and activate STING (93). STING is activated with a conformational change and is transported from the endoplasmic reticulum to the Golgi and forms oligomers (94, 95). Activated STING recruits TANK-binding kinase 1 (TBK1) and promotes its dimerization-mediated autophosphorylation, and activated TBK1, in turn, phosphorylates STING and further activates interferon regulatory factor 3 (IRF3) (96–98). Phosphorylated IRF3 forms dimers and ectopically translocates to the nucleus, where it acts in conjunction with NF-κB to induce the expression of type I interferons and inflammatory cytokines to initiate the innate immune response (99).

Emerging evidence suggests that the DNA-binding capacity or enzymatic activity of cGAS is affected by palmitoylation modifications (**Table 1**). Cytoplasmic DNA enhances ZDHHC18 interaction with cGAS, which promotes palmitoylation of cGAS on Cys474 and changes its conformation. cGAS conformational changes limit its enzymatic activity, which inhibits cGAS DNA binding and dimerization, although cGAS palmitoylation does not affect its subcellular localization (68). Therefore, the negative regulatory role of ZDHHC18-mediated modification of cGAS palmitoylation may be a novel regulatory mechanism in fine-tuning natural immunity (68). In contrast, ZDHHC9-mediated palmitoylation of cGAS at the 404/405 site is required for its dimerization and antiviral innate immunity signaling, and this process is inhibited by LYPLAL1 (69). It was found that dimerization mutants of cGAS (K394A or E398A) did not affect the level of palmitoylation, indicating that the palmitoylation modification is located upstream of the dimerization step. It is hypothesized that palmitoylation of cGAS may impact its signaling pathway in two ways: first, it may increase cGAS’s binding capacity to dsDNA to promote dimer formation, and second, it may palmitoylate cGAS to improve its dimerization upon DNA recognition (69). Although palmitoylation modifications have been shown to play a key regulatory role in cGAS dimerization, their detailed regulatory mechanisms need to be further investigated (**Figure 2**).

Although ZDHHC1 and ZDHHC11 both positively regulate DNA virus-triggered STING-dependent signaling, their methods of controlling STING activity differ: ZDHHC1 controls STING dimerization to enhance the subsequent recruitment and phosphorylation of TBK1 and IRF3 (100), and ZDHHC11 promotes the recruitment of IRF3 to STING to influence downstream signaling pathways (101). However, both palmitoyltransferase activities are not required for their regulation of STING-mediated signaling. Multiple studies have identified STING palmitoylation as a post-translational modification necessary for STING signaling. Palmitoylation modification of the Cys88/91 site of STING is necessary for STING-dependent IFN production in the trans-Golgi network (TGN) of the Golgi, but it does not affect its transport (70). Mechanistically, palmitoylation of STING activates the type I interferon signaling pathway by encouraging its aggregation in lipid rafts in the Golgi, which improves its interaction with downstream signaling molecules like TBK1 and IRF3 (70). However, palmitoylation of STING in endosomes can still be observed, suggesting that depalmitoylation of STING does not occur during transport from the Golgi to the degradation compartment (70). There is no evidence for the presence of depalmitoylating enzymes to depalmitoylate STING. Palmitylation-dependent STING clustering promotes TBK1 recruitment to STING. Subsequently, TBK1 phosphorylates STING, stabilizing TBK1 binding to the STING cluster. It establishes a positive feedback loop, maintaining the STING-TBK1 complex in a stable dynamic association (102). In recent years, several antagonists targeting STING palmitoylation have been developed to attenuate STING-mediated inflammation, such as C-170, H-151, nitrofatty acids (NO2-FAs), BPK-21, and 4-octyl itaconate, which block STING palmitoylation through covalent modification of Cys88/91 of SIING and inhibit its downstream type I interferon signaling (103–105). These findings demonstrate that STING palmitoylation is a potent pharmacological target for inhibiting STING signaling and thus for the treatment of STING-dependent inflammatory diseases.

### 3.4 NLRP3 inflammasome

As an important sensor in the innate immune system, the NLR family pyrin structural domain receptor 3 (NLRP3) recognizes PAMPs and DAMPs, and induces an inflammatory response through the formation of NLRP3 inflammasomes, which eliminate invading pathogens and repair damaged tissues, restoring homeostasis in the body (106–108). NLRP3 is an important PRR in the cytoplasm and consists of a pyridine structural domain (PYD), a NACHT structural domain for nucleotide binding and ATPase activity, and a leucine-rich repeat (LRR) structural domain (109, 110). Activation of NLRP3 inflammatory vesicles includes classical, nonclassical, and alternative pathways (111). There are two steps in the NLRP3 inflammatory vesicle’s classical activation pathway. During the initiation phase, PRR recognition of PAMPs and DAMPs activates the NF-κB signaling pathway and promotes the upregulation of NLRP3, pro-IL-1β, and pro-IL-18 gene expression (112). During the activation phase, stimuli such as Nigericin (113) prompted NLRP3 to oligomerize through the NACHT structural domain, recruit apoptosis-associated speck-like protein containing a CARD (ASC) and NIMA-associated kinase 7 (NEK7), and promote ASC oligomerization to form macromolecular foci of specks, which in turn led to pro-caspase-1 recruitment and activation, and assembly to form NLRP3 inflammatory vesicles (114). Activated caspase-1 cleaves pro-IL-1β and pro-IL-18 to generate mature IL-1β and IL-18 while cleaving gasdermin-D (GSDMD) and triggering pyroptosis (115).

**Figure 3:**
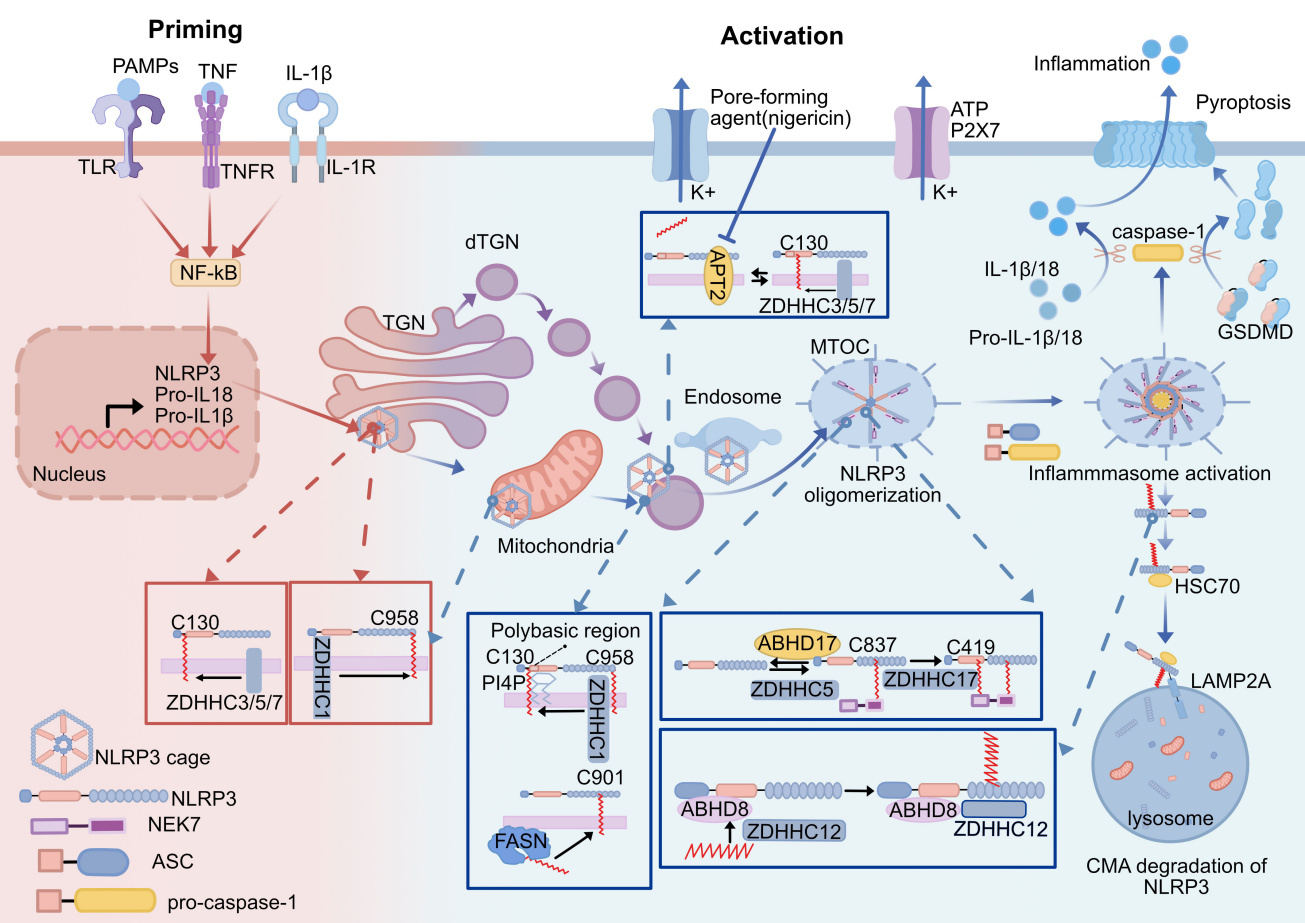
Effect of the palmitoylation of NLRP3 inflammasome. Activation of NLRP3 inflammasome is divided into priming and activation stages. (1) ZDHHC1 mediates sequential palmitoylation of NLRP3 at C958 and C130 during priming and activation, directing NLRP3 to target specific membranes(TGN, mitochondria, endosome)and ultimately to MTOC. (2) ZDHHC3/5/7-mediated NLRP3 Cys130 palmitoylation promotes resting NLRP3 localizing on TGN and activated NLRP3 on the dTGN. The NLRP3 Cys 130 palmitoylation modification is able to be removed by APT2 to reduce NLRP3 binding to the Golgi. Nigericin increases the level of palmitoylation of NLRP3 by altering the Golgi organization and function to localize APT2 in the Golgi. The polybasic region of NLRP3 interacts with PI4P to assist palmitoylation to achieve stable membrane binding of NLRP3. (3) Palmitoylation modification of the NLRP3 Cys901 site translocates NLRP3 to dTGN, which is dependent on FASN-mediated fatty acid synthesis. (4) NLRP3 palmitoylation at Cys 837/838 and C419 promotes NLRP3 binding to NEK7 and NLRP3 oligomerization. (5) ZDHHC12-mediated palmitoylation of NLRP3 promotes NLRP3 degradation through the chaperone-mediated autophagy pathway, and ABHD8 acts as a scaffold to recruit ZDHHC12 to NLRP3. ZDHHC, zinc finger DHHC domain-containing protein; APT2, Acyl-Protein Thioesterase 2; ABHD, α/β-hydrolase domain-containing; TLR, toll-like receptor; PAMPs, pathogen-associated molecular patterns;TNF, tumor necrosis factor; TGN, trans-Golgi network; dTGN, dispersed trans-Golgi network; NEK7, NIMA-related kinase 7; ASC, apoptosis-associated speck-like protein containing a CARD; PI4P, phosphatidylinositol-4-phosphate; MTOC, microtubule-organizing center; GSDMD, gasdermin-D; HSC70, heat shock cognate protein of 70 kDa; LAMP2A, lysosome-associated membrane protein 2A; CMA, chaperone-mediated autophagy.

Multiple NLRP3 palmitoylation sites catalyzed by distinct ZDHHCs significantly modulate the activity of inflammatory vesicles by precisely ordered regulation of their localization and stability at different stages of inflammasome assembly and activation (**Table 1**). Notably, membrane trafficking is essential for the assembly and activation of the NLRP3 inflammasome, and palmitoylation modifications can regulate protein transport and membrane binding (116). Palmitoylation of Cys130, 901, 958 in human NLRP3 and the corresponding Cys126/898/955 in mice has been found to regulate the mechanism of NLRP3 targeting the Golgi at different stages of the process (71–73, 117). During the initiation phase, the bicyclic cage structure of NLRP3 keeps it inactive (118), and NLRP3 can be recruited to the TGN through its highly conserved polybasic region interacting with the negatively charged phosphatidylinositol 4-phosphate (PtdIns4P) on the TGN, while the polybasic region alone is not sufficient to achieve stable membrane binding, and coactivation with palmitoylation is required to stabilize the membrane localization of NLRP3 (117). Palmitoylation of NLRP3 on both Cys130 and Cys958 promotes resting-state NLRP3 localization to the TGN, in which ZDHHC1-mediated palmitoylation modification of C958 enhances the affinity of the inactive NLRP3 cage structure and facilitates its TGN localization (73). Yu et al. demonstrated that in mouse macrophages, Cys126 is located in the junction region between the Pyrin and NACHT structural domains of the NLRP3 protein and is adjacent to a polybasic region involved in the regulation of NLRP3 TGN localization. The ZDHHC7 palmitoylates NLRP3 on Cys126, and thus mutation of NLRP3 Cys126 would disrupt its localization in the TGN as well as the recruitment and oligomerization of ASCs, inhibiting the activation of the inflammasome (71) (**Figure 3**).

During the activation phase, NLRP3 is transiently attached to mitochondria, followed by structural dissociation of the TGN to form a dispersed TGN (dTGN). dTGN can transport NLRP3 to the microtubule organizing center (MTOC), where NLRP3 recruits NEK7 and ASC and assembles the inflammasome (119–121). The transient mitochondrial localization of NLRP3 in the early stages of activation requires its palmitoylation modification at Cys958. Nevertheless, after mitochondrial localization, it is the Cys130 palmitoylation that is required for PI4P binding and translocation of NLRP3 to PI4P-rich membranes such as the dTGN and endosomes (73). In addition, NLRP3 achieves dynamic binding to the Golgi apparatus at Cys130 through a palmitoylation cycle, and the dynamic balance between palmitoylation (catalyzed by ZDHHC3/7) and depalmitoylation (mediated by APT2) regulates the residence time of NLRP3 at the membrane (72). When the organism is in homeostasis, NLRP3 is maintained in a low activity state through the circulation; once stress disrupts the homeostasis, damage to the Golgi structure decreases APT2 contact with the Golgi, which leads to retention of palmitoylated NLRP3 in the Golgi and overactivation of the inflammasome (72). FASN depletion inhibits NLRP3 Cys898 palmitoylation in mice, suppressing its dTGN localization. It reveals lipid metabolism’s role in NLRP3 activation (117).

NEK7 is a critical coactivator in NLRP3 inflammasome assembly and is a core component specific to the NLRP3 inflammasome (122, 123). Interaction between NLRP3 and NEK7 is affected by palmitoylation. Both *in vitro* and *in vivo* studies show that ZDHHC5 palmitoylates Cys837/838 in the NLRP3 LRR structural domain, promoting NLRP3-NEK7 interactions. ZDHHC5 knockdown inhibits NLRP3 oligomerization, NLRP3-NEK7 interactions, and the formation of intracellular ASC macroaggregates, reducing mice’s inflammatory response (74). Palmitoylation has a regulatory role in the stability of NLRP3.

ZDHHC12 is a negative regulator of NLRP3 activation, which catalyzes palmitoylation of NLRP3 at Cys844 (the corresponding site of mouse NLRP3 C841), thereby facilitating recognition of NLRP3 by heat shock cognate protein of 70 kDa (HSC70) to promote NLRP3 degradation via the chaperone-mediated autophagy (CMA) pathway, which prevents sustained inflammation. Unlike other NLRP3 modifications, ZDHHC12-mediated palmitoylation modification occurs late in the completion of inflammasome function, which acts as a brake to shut down the inflammasome (76). ABDH8 was recently discovered to function as a scaffold to attract ZDHHC12 to NLRP3, promoting NLRP3 palmitoylation and the subsequent CMA-mediated degradation of NLRP3, although it lacks an acyltransferase active motif (124). In addition, inhibition of palmitoylation similarly inhibits non-classical NLRP3 inflammasome activation, but the exact mechanism is unknown (73). NLRP3 phase separation is an essential prerequisite for its activation. In resting cells, ZDHHC7-mediated constitutive palmitoylation of NLRP3 lowers the threshold for phase separation, thereby enabling cellular responsiveness to diverse stimuli (including intracellular perturbations and NLRP3-binding molecules) to trigger NLRP3 activation. In contrast, ABHD13 antagonistically modulates this process (75).

### 3.5 Gasdermin-mediated pyroptosis pathway

Pyroptosis is essential for both host defense and the etiology and pathophysiology of inflammatory diseases (125). Cellular pyroptosis is mediated by the Gasdermin (GSDM) family of proteins that form membrane pores (126). GSDMA, GSDMB, GSDMC, GSDMD, GSDME, and DFNB59 are the six members of the GSDM protein family (127). Except for DFNB59, all GSDMs contain two conserved structural domains: a C-terminal containment domain (CTD) and an N-terminal structural domain (NTD) (128).

**Figure 4:**
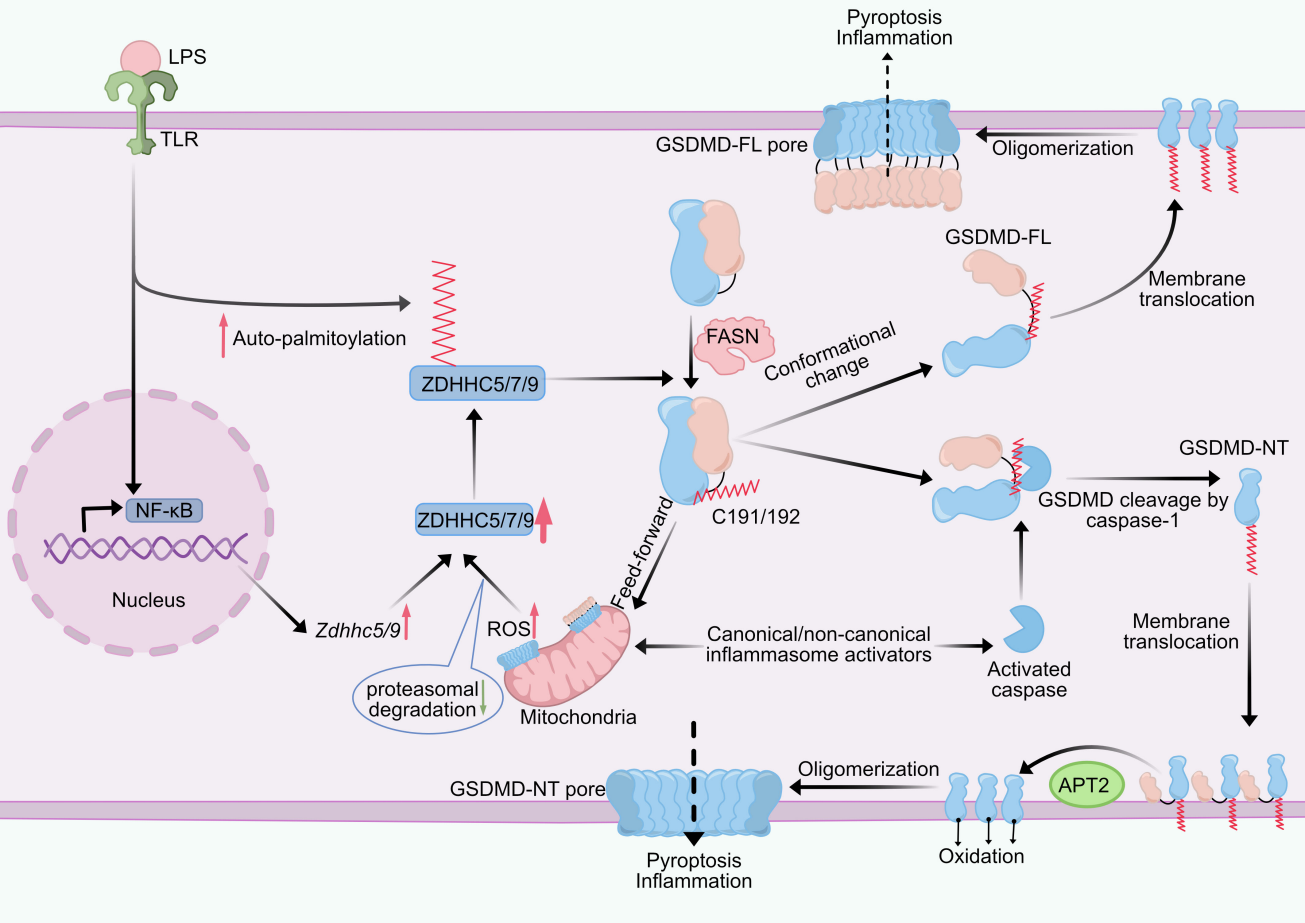
Effect of palmitoylation on GSDMD. GSDMD is palmitoylated at Cys191/192 (human/mouse). Upon LPS stimulation, the NF-κB pathway and ZDHHC auto-palmitoylation levels are upregulated, leading to increased expression levels of ZDHHC5/7/9 and enhanced GSDMD palmitoylation. There is a feed-forward loop between reactive oxygen species (ROS) and GSDMD palmitoylation. Palmitoylated GSDMD damages mitochondria, leading to ROS production, which enhances GSDMD palmitoylation by reducing proteasomal degradation caused by ZDHHC enzymes. GSDMD palmitoylation directs its cleavage by caspases, GSDMD-NT palmitoylation promotes its translocation to the plasma membrane, APT2 depalmitoylates GSDMD-NT at the plasma membrane and promotes GSDMD oligomerization, and ROS-mediated oxidation of C192 residues together with palmitoylation mediates GSDMD-NT oligomerization. GSDMD palmitoylation also induces a conformational change in GSDMD, releasing GSDMD-CT autoinhibition and activating full-length GSDMD, which causes less efficient pyroptosis than GSDMD-NT. ZDHHC, zinc finger DHHC domain-containing protein; APT2, acyl protein thioesterase 2; GSDMD, gasdermin D, GSDMD-NT, N-terminal domain; GSDMD-FL, full-length GSDMD; LPS, lipopolysaccharide; TLR, toll-like receptor; ROS, reactive oxygen species; FASN, fatty acid synthase.

GSDMD is essential for host defense against pathogen infection as a key execution protein for inflammasome-induced cellular pyroptosis (129). Upon its activation, it is cleaved by inflammatory caspases (130), and the released N-terminal fragment (GSDMD-NT) oligomerizes to form a pore in the plasma membrane. This enables the release of pro-inflammatory cytokines like IL-1β and IL-18 and damages the integrity of the cell membrane, which causes inflammation and pyroptosis (131, 132). GSDMD is central to pyroptosis-induced inflammatory injury, so studies on the mechanisms regulating the conformation, transport, and pore-forming activity of GSDMD proteins are important for therapeutic targeting of inflammatory diseases (133). Palmitoylation has been identified as a key regulatory mechanism controlling GSDMD membrane localization and activation (**Table 2**). Recent research has demonstrated that GSDMD-mediated cellular pyroptosis also involves palmitoylation modification of Cys191/192(mouse/human). ZDHHC7 and ZDHHC5/9 were found to mediate palmitoylation of Cys191/192, which is explained by the enzymes’ intrinsic redundancy and broad substrate specificity (78, 79). Palmitoylation was found to affect pyroptosis by regulating GSDMD processing and membrane translocation. Specifically, GSDMD palmitoylation was able to promote its interaction with caspase-1 to increase GSDMD processing, while palmitoylation was able to promote plasma membrane ectopic translocation of GSDMD-NT during the process of colocalization (78). Furthermore, this study also found that the level of ZDHHC7 autopalmitoylation, which mediates GSDMD palmitoylation, was elevated, suggesting that LPS may induce GSDMD palmitoylation through this mechanism (78). Interestingly, APT2 is critical for GSDMD pore formation. APT2 depalmitoylates GSDMD-NT at the membrane to reveal Cys192 residues, thereby promoting GSDMD-NT oligomerization and pyroptosis (78). Intervention of palmitoyltransferase inhibitor 2-BP in LPS-induced mice significantly inhibits inflammation-induced tissue damage, reduces pyroptosis, and improves mouse survival (79). Notably, in contrast to the notion that division is the only trigger for GSDMD activation, full-length GSDMD (GSDMD-FL) is also capable of inducing liposome leakage by palmitoyl modification and forming a pore structure similar to that of the GSDMD-NT pore and triggering pyroptosis. However, GSDMD-FL-mediated pyroptosis was not as efficient as that of GSDMD-NT, and full-length GSDMD could not induce pore formation and pyroptosis if it was not palmitoylated (134). These findings demonstrate that GSDMD palmitoylation is required for pore formation (**Figure 4**).

FASN is a binding chaperone for GSDMD, and the interaction between FASN and GSDMD is only observed in LPS-stimulated macrophages (135). As a key regulator of inflammatory cell death, ROS have recently been found to affect GSDMD’s palmitoylation through various mechanisms. A feed-forward loop exists between GSDMD palmitoylation and ROS (134). Palmitoylated GSDMD-FL/NT is recruited to the mitochondria to form a pore, which increases ROS production, and high ROS in turn increases ZDHHC5 and ZDHHC9, leading to more GSDMD palmitoylation. However, ROS alone is not sufficient to cause enhanced GSDMD palmitoylation in the absence of LPS stimulation (135). It was demonstrated that inhibition of NF-κB activation significantly inhibits LPS-induced GSDMD palmitoylation, and ROS scavengers in turn, inhibit NF-κB activation; thus, the enhancement of GSDMD palmitoylation by ROS is partly due to NF-κB activation (135).

In addition to GSDMD, other mammalian gasdermin family members, GSDMA, GSDMB, GSDMC, and GSDME, may also undergo palmitoylation at the NT structural domain (79). Palmitoylation of the C-terminus of GSDME (GSDME-CT) is involved in the process of chemotherapy-induced pyroptosis. This palmitoylation event was able to hinder the interaction of GSDME-NT and GSDME-CT and promote their dissociation, leading to an increase in chemotherapeutic drug-induced pyroptosis, and this study provides a new target for achieving the transition between chemotherapy-induced pyroptosis and apoptosis (80). Palmitoylation plays a key regulatory role in pyroptosis and can provide new therapeutic avenues for pyroptosis-related diseases (136).

### 3.6 Cross-pathway coordination

Palmitoylation, as an important post-translational modification of proteins, plays a finely regulated role in the whole process of inflammatory signaling pathways through its dynamic and reversible properties. In this review, we found that different ZDHHC family members constitute a multilevel inflammatory regulatory network through a spatiotemporally specific substrate recognition mechanism. Specifically, ZDHHC6 significantly promotes the activation initiation of the TLR signaling pathway by mediating the palmitoylation modification of MYD88 and TLR2, whereas ZDHHC3/5/7 are involved in the activation phase of the inflammatory response by regulating the membrane localization and conformational changes of effector molecules such as STING, NLRP3, and GSDMD. Particularly noteworthy, the present study revealed that ZDHHC12 plays a critical negative regulatory role in the regressive phase of inflammation by promoting the degradation of NLRP3. This synergistic effect of positive activation and negative regulation constitutes a complete inflammatory “initiation-activation-abatement” regulatory loop. Further mechanistic studies suggest that palmitoylation modifications achieve precise spatiotemporal regulation of inflammatory responses by affecting the subcellular localization, protein stability, and molecular interactions of key inflammatory proteins. These findings not only deepen our understanding of the role of protein palmitoylation in natural immunity but also provide a new theoretical basis and therapeutic targets for the development of ZDHHC-based intervention strategies for inflammatory diseases.

## 4 Palmitoylation and inflammatory diseases

### 4.1 Inflammatory bowel disease

Inflammatory bowel disease (IBD) is a chronic, recurrent inflammatory disease of the gastrointestinal tract, which is divided into two main subtypes: ulcerative colitis (UC) and Crohn’s disease (CD) (137, 138). Despite the continued rise in the incidence of IBD, the complex molecular and cellular mechanisms underlying the pathogenesis of IBD remain poorly understood, and the treatment of IBD is poorly effective (139). The pathophysiology of IBD is multifactorial and involves a complex interplay of genetic, environmental, epithelial, microbial, and immunologic factors (140, 141).

Emerging evidence highlights the therapeutic potential of targeting protein palmitoylation in IBD (**Table 3**). In IBD patients, elevated ZDHHC7 and APT2 levels correlate with enhanced STAT3 palmitoylation and Th17 hyperactivity. Animal experiments show that the knockdown of *Zdhhc7* or inhibition of APT2 to interrupt the palmitoylation-depalmitoylation cycle alleviates the symptoms of colitis in a mouse model, suggesting that the STAT3 palmitoylation cycle and its regulatory enzymes may be new therapeutic targets for colitis (142). Li et al. explored the mechanism of treating IBD by targeting the STAT3 palmitoylation cycle. 2’-Fucosyllactose inhibits STAT3-related signaling pathways in colonic tissue by suppressing STAT3 phosphorylation and palmitoylation, ultimately repairing the intestinal mucosal barrier in ulcerative colitis and reducing inflammatory responses (143). Long-chain fatty acids (LCFAs) can enter intestinal epithelial cells through CD36-mediated endocytosis and participate in the palmitoylation cycle of STAT3. This process not only promotes STAT3 phosphorylation and nuclear translocation but also aggravates intestinal inflammatory response and intestinal barrier damage, Therefore, controlling the intake of LCFAs is a potential strategy for the prevention and treatment of IBD (144). Notably, pharmacological inhibition of ZDHHC7 and APT2 remains an unexplored therapeutic strategy for IBD, despite their potential as novel drug targets in IBD treatment.

**Table 3:** Aberrant palmitoylation in disease-related proteins and their regulatory enzymes and pathological mechanisms.

Disease	Aberrant Protein	Regulatory enzymes	Pathological Mechanism	References
IBD	STAT3	ZDHHC7, APT2	The palmitoylation cycle in STAT3 enhances its membrane localization and phosphorylation and promotes Th17 cell differentiation	(142–144)
IBD	Mucin 2	FASN	Reduced palmitoylation of Mucin2 causes impaired mucus secretion, abnormal intestinal barrier permeability, and flora-immunity imbalance	(145)
IBD	Akt	FASN	Akt palmitoylation activates the FASN/Akt/p65 pathway and increases pro-inflammatory responses in macrophages	(146)
IBD	GSDMD	FASN	Increased GSDMD palmitoylation promotes pyroptosis and exacerbates intestinal inflammatory responses	(147)
IBD	–	ZDHHC6	Increased levels of ZDHHC6 are positively associated with disease progression in inflammatory bowel disease	(148)
IBD	NOD2	ABHD17A, B, C	Reduced palmitoylation of NOD2 leads to abnormalities in its signaling, contributing to reduced NF-κB and pro-inflammatory cytokine production in epithelial cells	(149)
Systemic lupus Erythematosus (SLE)	TLR9	ZDHHC3, PPT1	PPT1 promotes the secretion of IFNα by plasmacytoid dendritic cells (pDCs)and TNF by macrophages, raises antinuclear antibodies and increases severity of nephritis	(60)
SLE	MAVS	ZDHHC12	MAVS palmitoylation causes its aberrant aggregation in macrophages, reduces type I interferon response	(150)
Psoriasis	Unknown	ZDHHC2	ZDHHC2 reduction inhibits pDC accumulation and activation in psoriatic skin, spleen and draining lymph nodes	(151)
Multiple sclerosis	Mothers against decapentaplegic homolog 2(SMAD2)	ZDHHC7, APT2	The SMAD2 palmitoylation cycle drives Th17 cell differentiation by enhancing phosphorylation of its linker region and promoting interaction with STAT3	(152)
MASH	CD36	ZDHHC4,5,7	CD36 hyper-palmitoylation enhances fatty acid uptake, driving mitochondrial β-oxidation overload, lipid accumulation, ROS overproduction, oxidative stress, and hepatic inflammation.	(153–155)
MASH	Akt	ZDHHC17, 24/APT2	Akt palmitoylation is important for the PI3K-Akt signaling pathway and promotes NASH formation	(156)
MASH	RIPK1	ZDHHC5	DHHC5 is amplified by fatty acids and promotes RIPK1 cytotoxicity and liver injury in MASH	(157)
MASH	IRHOM2	ZDHHC3	IRHOM2 palmitoylation decreases ubiquitinated degradation of IRHOM2 and promotes its activation and downstream MAP3K7-JNK signaling, leading to increased hepatic steatosis, inflammation, and collagen accumulation	(158)
MASH	STING	–	STING palmitoylation enhances STING transport to the Golgi, activates downstream signaling, and induces pro-inflammatory and pro-fibrotic cytokine secretion, resulting in hepatic steatosis and hepatic stellate cell activation	(159)
MASH	AEG-1	ZDHHC6/ PPT2	Reduced AEG-1 palmitoylation activated proliferation, migration, inflammatory response, angiogenesis, and lipid accumulation in hepatocytes, enhancing their oncogenic potential, reduced the activation of pro-inflammatory and pro-MASH pathways in periportal and pericentral hepatocytes, inhibited xenobiotic metabolism (XM) pathways in mid-lobular zone	(160, 161)
Sepsis	Unknown	ZDHHC21	Increased ZDHHC21 reduces complement enrichment in extracellular vesicles and promotes neutrophil adhesion, migration and neutrophil extracellular trap production	(162)
Sepsis	STING	–	STING palmitoylation promotes STING-STXBP2 interaction and exacerbates septic thrombosis by triggering SNARE complex formation, granule secretion and platelet activation	(163)
Sepsis	CD36	–	CD36 depalmitoylation causes it to move to the lysosomal membrane, causing impaired autophagosomal-lysosomal fusion and liver injury in sepsis	(164)
Sepsis	NLRP3	ZDHHC12	NLRP3 palmitoylation activates the inflammasome, exacerbates oxidative stress and inflammation, and promotes exacerbation of septic myocardial injury	(165)
Sepsis	α1AR	ZDHHC21	α1AR palmitoylation leads to renal artery vasoconstriction, causing impaired renal function and perfusion in septic injury	(166)
Alzheimer’s disease	Beclin 1	ZDHHC5	Decreased DHHC5 in the brain leads to reduced neuronal autophagy and promotes Aβ-induced neurodegeneration, long-term potentiation deficits, and memory deficits	(167)
Alzheimer’s disease	Fyn	ZDHHC21	ZDHHC21 p.T209 Significantly increases Fyn palmitoylation, which leads to synaptic damage, excitotoxicity and neuronal cell injury	(168)
Alzheimer’s disease	BACE1	ZDHHC7	BACE1 palmitoylation increases BACE1 neuron transport and accumulation in dystrophic synapses near AD brain amyloid deposition, promoting synaptic activity-induced Aβ production, increasing amyloid loading, and memory deficits	(169)
Alzheimer’s disease	APH1 and Nicastrin	–	Palmitoylation increases Aβ levels and amyloid deposits in the brain	(170)
Alzheimer’s disease	APP	–	APP palmitoylation promotes its targeting to lipid rafts and enhances amyloid processing through enhanced BACE1-mediated cleavage	(171)
Alzheimer’s disease	TRPV2	ZDHHC21	TRPV2 palmitoylation reduces microglia Aβ phagocytosis	(172)
Alzheimer’s disease	CD36	ZDHHC6	CD36 palmitoylation reduces the localisation of CD36 in microglia membranes and inhibits Aβ phagocytosis	(173)
Alzheimer’s disease	PrRP		Reduced PrRP palmitoylation significantly increases Aβ plaque loading and microglia proliferation in the cerebellum and reduces synaptogenesis to promote neuroinflammation and apoptosis	(174)
Alzheimer’s disease	PSD-95		Decreased PSD-95 palmitoylation intensifies Aβ’s detrimental impact on synapses	(175, 176)
Parkinson’s disease	Syt11	–	Syt11 palmitoylation promotes membrane binding of alpha-synuclein and pathological monomer aggregation	(177)
Parkinson’s disease	MAP6	APT1	Palmitoylation of MAP6 increases αS phosphorylation, inclusion bodies, and cytotoxicity	(178)
Parkinson’s disease	estrogen receptor alpha (ERα)	–	Reduced ERα palmitoylation decreases αS solubility, which in turn impairs synaptic plasticity and motor and cognitive phenotypes	(179)
Diabetes Mellitus	eNOS	FASN	Reduced eNOS palmitoylation leads to impaired inflammation and angiogenesis by causing endothelial dysfunction	(180)
Diabetic neuropathy	PRDX6	–	PRDX6 palmitoylation improves interaction with anion exchanger 3 (AE3), triggering Cl−/HCO_3_− ion flow, causing pain in the dorsal root ganglion (DDRG)	(181)
Diabetic neuropathy	PEX11B	–	PEX11B palmitoylation interferes with PEX11B self-interactions and prevents peroxisome fission, leading to peroxisome dysfunction in the Schwann cell	(182)
Diabetic retinopathy	SMPDL3B	ZDHHC5	Increased palmitoylation of SMPDL3B reduces its degradation and alleviates diabetic retinopathy by inhibiting the NF-κB/NLRP3 pathway	(183)
Diabetic kidney disease	R-Ras	APT1	R-Ras palmitoylation constrains R-Ras membrane trafficking constrained R-Ras membrane trafficking impairs fibronectin processing and reduces adhesion junctions, leading to defects in APT1-deficiency-induced lumen formation	(184)
Diabetic cardiomyopathy	CD36	ZDHHC4	CD36 palmitoylation enhances cardiac fatty acid uptake and leads to lipid accumulation	(185)
Diabetic foot ulcers	NLRP3	PPT1	NLRP3 palmitoylation drives pro-inflammatory macrophage phenotypes	(77)

ZDHHC, zinc finger DHHC domain-containing protein; APT, acyl protein thioesterase; FASN, fatty acid synthase; PPT, palmitoyl-protein thioesterase; STAT3, signal transducer and activator of transcription 3; GSDMD, gasdermin D; PPARγ, peroxisome proliferator-activated receptor gamma; NOD2, nucleotide-binding oligomerization domain-containing proteins2, TLR9, toll-like receptor 9; MAVS, mitochondrial antiviral-signaling protein; Akt, Ak strain transforming;RIPK1, receptor-interacting serine/threonine protein kinase 1; IRHOM2, inactive rhomboid protein 2; STING, stimulator of interferon genes; AEG-1, astrocyte-elevated gene-1; α1AR, α1-adrenergic receptor; STXBP2, syntaxin binding protein 2; BACE1, β-Site APP cleaving enzyme 1; Aβ, β-Amyloid peptides; APP, amyloid precursor protein; ACAT, acyl-coenzyme A:cholesterol acyltransferase; TRPV2, transient receptor potential vanilloid 2; PrRP, prolactin-releasing peptide; Glut4, glucose transporter 4; eNOS, endothelial nitric-oxide synthase; SMPDL3B, peroxiredoxin-6; Sphingomyelin phosphodiesterase acid-like 3B; MAP6, microtubule-associated-protein-6; IBD, inflammatory bowel disease; SLE, systemic lupus erythematosus; MASH, metabolic dysfunction-associated steatohepatitis; Syt11, Synaptotagmin-11.

FASN can influence protein palmitoylation to participate in the inflammatory response and mucosal barrier impairment in IBD. Colonic epithelial cell-specific knockdown of the FASN gene significantly inhibits Mucin 2 (MUC2) palmitoylation, leading to impaired mucus secretion, abnormal intestinal barrier permeability, and flora-immunity imbalance, which eventually may induce colitis and systemic inflammatory response (145). Metformin inhibits Akt palmitoylation through FASN downregulation, which prevents its membrane translocation and activation, consequently blocking MAPK-mediated inflammatory signaling in LPS-induced macrophages. In a mouse model of colitis, metformin effectively inhibited the pro-inflammatory response of colonic intrinsic monocytes through this mechanism to alleviate colitis (146).

Palmitoylation of pyroptosis-related proteins is likewise closely associated with the onset and progression of IBD. NU6300 improved the pathology of DSS-induced acute colitis, GSDMD cleavage, and inflammatory cytokine release. Mechanistically, NU6300 forms a covalent reaction with the C191 residue of GSDMD, inhibiting pyroptosis by blocking its cleavage and palmitoylation. However, NU6300 has potential cardiac toxicity and low blood exposure levels, necessitating further structural optimization of the drug in the future (147). Existing GSDMD covalent inhibitors (disulfiram, necrosulfonamide, dimethyl fumarate) all target Cys191, but their impact on palmitoylation is unknown, highlighting the potential of developing palmitoylation-site-specific modulators for IBD (186–188). Inflammasome activation and the progression of inflammatory bowel disease are modulated by palmitoylation of NLRP3, while inflammasome activation and the severity of dextran sulfate sodium (DSS)-induced colitis in mice are effectively attenuated by pharmacological inhibition of NLRP3 palmitoylation (20).

Some palmitoylation-related enzymes are important regulators of IBD; however, the exact mechanism of their association with palmitoylation is unclear. ZDHHC6 levels are significantly elevated in the colonic tissues of patients with clinical IBD and correlate with diagnostic indicators of colitis (ESR, CRP, ALB). In the DSS-induced colitis model, ZDHHC6 expression increased with disease progression, suggesting that elucidating the precise molecular interactions and pathways mediated by ZDHHC6-mediated palmitoylation regulation will be an important research direction for the development of IBD-targeted therapies (148). ABHD17 inhibition significantly promotes the plasma membrane localization of NOD2 in palmitoleic acid-deficient Crohn’s disease-associated mutants (R702W, L248R, and A755V), enhancing their functional activity. This regulatory effect restores the activation of the NF-κB signaling pathway in intestinal epithelial cells mediated by mutant NOD2 and promotes the production of proinflammatory cytokines (149).

### 4.2 Autoimmune diseases

Autoimmune diseases (AIDs) are a group of disorders in which the immune system develops an immune response to its own normal tissue components (189), characterized by chronic, systemic, and excessive immune activation and inflammation, ultimately leading to organ destruction or dysfunction (190). Environmental factors, genetic factors, and unusual infections often lead to the induction of AIDs (191, 192). Around 100 AIDs have been identified, and common AIDs include rheumatoid arthritis, systemic lupus erythematosus (SLE), psoriasis, and multiple sclerosis.

Palmitoylation modifications play a key role in the development of autoimmune diseases and can modulate immune and inflammatory signaling pathways in these diseases (**Table 2**). PPT1 promotes pDC secretion of IFNα and macrophage secretion of TNF by regulating TLR9 depalmitoylation. In SLE models and patients, the PPT1 inhibitor HDSF significantly reduces IFNα levels and autoantibody production. Notably, HDSF exhibits a unique dual action by suppressing TLR signaling in pDCs to alleviate autoimmunity while enhancing CTL responses in cDC1 to maintain antitumor/anti-infective capacity. In contrast, the ZDHHC3 inhibitor 2-BP, though capable of inhibiting IFNα and immune cell proliferation *in vitro*, lacks this cellular selectivity. These findings suggest that targeting PPT1 may represent a potential therapeutic strategy for SLE (60, 193). The palmitoylation modification of the mitochondrial antiviral-signaling protein (MAVS) protein is a key regulatory step in the activation of the RLR (RIG-I-like receptor) signaling pathway and the IFN-I response. Pothlichet et al. found that African American SLE patients had the MAVS-C79F mutation, and patients with this mutation showed significantly lower IFN-α levels (194). Wang et al. recently demonstrated that the C79F mutation disrupts the palmitoylation site of MAVS, leading to oligomerization defects and significantly weakening the RLR pathway-induced IFN-I response (150). Existing studies have shown that multiple palmitoylation regulatory molecules (ZDHHC7, ZDHHC24, ZDHHC4, and APT2) can influence the palmitoylation status of MAVS, affecting its mitochondrial localization and signal transduction. However, the patterns of MAVS palmitoylation changes in SLE and their regulatory mechanisms remain unclear, necessitating further investigation into the expression changes of these enzymes in SLE patients and their regulatory mechanisms on MAVS activity (195–197).

ZDHHC2 deficiency attenuates pathological progression in mice by inhibiting pro-inflammatory cytokine expression in inflamed skin and leukocyte infiltration in psoriatic lesion areas, and ZDHHC2 deficiency reduces migration of pDC to the skin and other organs, which reduces psoriasis risk (151). In addition, Ni et al. confirmed that TLR7 can undergo palmitoylation modification (60). Given that abnormal activation of TLR7 has been clearly implicated in the pathogenesis of psoriasis, this post-translational modification may become a new target for psoriasis research by regulating the activity of the TLR7 signaling pathway (198). ZDHHC7 and APT2 promote the formation and nuclear translocation of the SMAD2-STAT3-SMAD4 complex by regulating the palmitoylation-depalmitoylation dynamic balance of SMAD2 and STAT3, thereby driving the differentiation of naive T cells into Th17 cells. In the multiple sclerosis model, the absence of ZDHHC7 or APT2 leads to reduced Th17 cells, increased Treg cells, and significantly alleviated central nervous system inflammatory infiltration and demyelination damage. This finding reveals that targeting the SMAD2/STAT3 palmitoylation cycle can inhibit Th17 differentiation, providing a new therapeutic strategy for multiple sclerosis (152).

### 4.3 Metabolic dysfunction-associated steatohepatitis

Metabolic dysfunction-associated steatotic liver disease (MASLD) is the most common chronic liver disease (199). It is a progressive disease that starts with simple steatosis (non-alcoholic fatty liver, or NAFL) and can develop into a more complex form called metabolic dysfunction-associated steatohepatitis(MASH) (200). MASH is characterized by liver inflammation and hepatocyte damage (ballooning) due to fat accumulation, which together lead to fibrosis (201, 202). MASH has become the main cause of end-stage liver disease and liver transplantation. Currently, there is no approved effective treatment for the disease, leading to an increasing socioeconomic burden and a serious impact on the quality of life of patients (203, 204). Therefore, more extensive research is necessary to reveal the pathogenesis of MASH and to provide more effective treatments for MASH.

**Figure 5:**
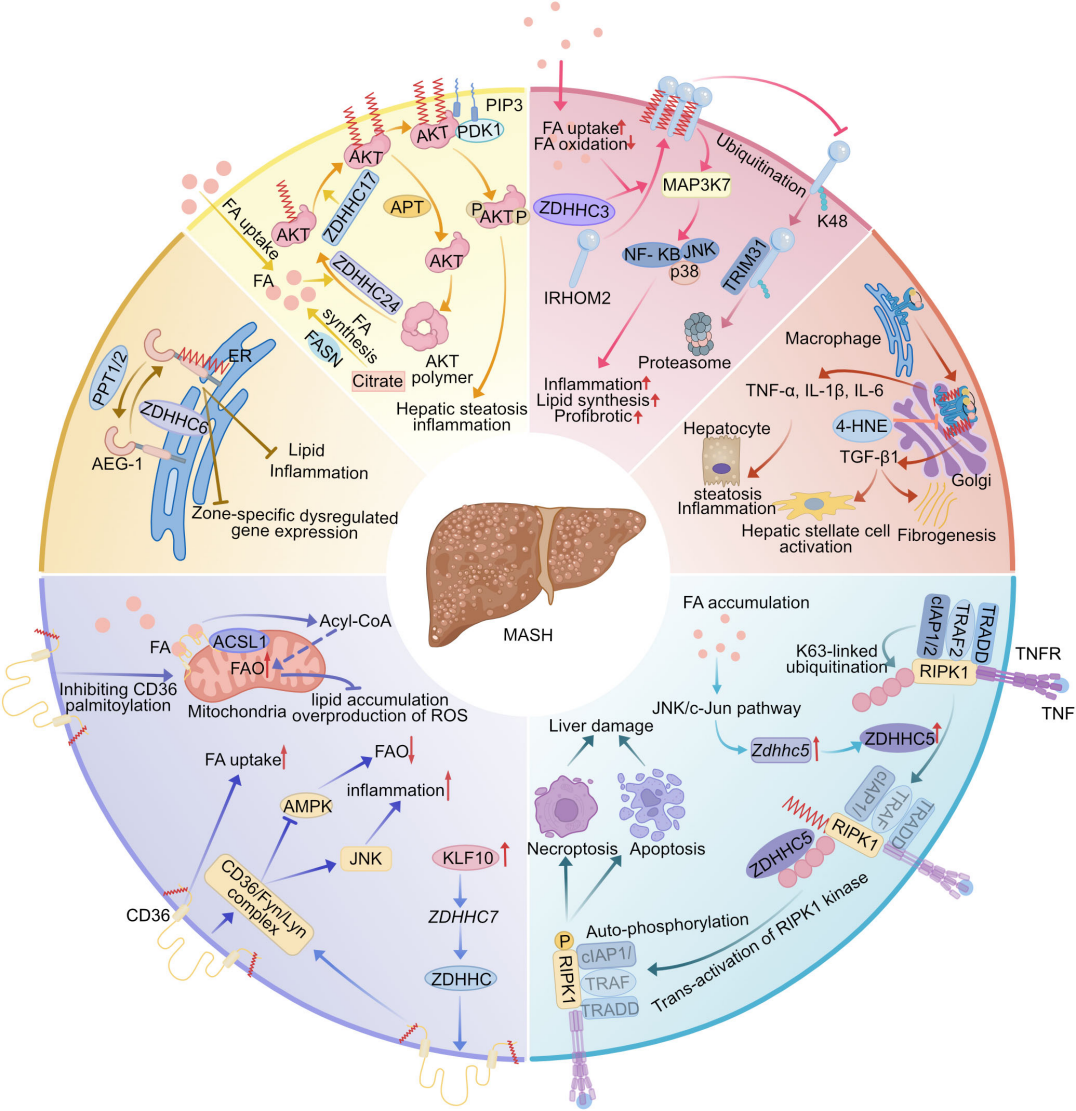
Multiple regulation of the pathogenesis of MASH by palmitoylated proteins. (1) CD36 palmitoylation increases CD36 plasma membrane localization, which leads to increased FA uptake, decreased FAO, and activation of inflammatory responses by promoting the formation of the CD36/Fyn/Lyn complex, while KLF10 transcriptionally activates ZDHHC7 to promote CD36 palmitoylation. Inhibition of CD36 palmitoylation translocates it to the mitochondrial membrane to interact with ACSL1 and enhance hepatic fatty acid β-oxidation. (2) AEG-1 palmitoylation inhibits hepatic lipid accumulation and inflammation, and inhibition of its palmitoylation leads to dysregulation of gene expression in different regions of the liver, which impairs hepatic metabolic homeostasis. (3) Palmitoylation anchors AKT to the cell membrane, in part by preventing the assembly of AKT into inactive polymers, increasing AKT activation and promoting MASH. (4) IRHOM2 palmitoylation promotes its translocation across the cell membrane and inhibits IRHOM2 ubiquitin-proteasome-associated degradation mediated by TRIM31. Fatty acid treatment enhances IRHOM2 palmitoylation by increasing direct binding between ZDHHC3 and IRHOM2, promoting hepatic steatosis and inflammation. (5) STING palmitoylation activates its downstream signaling pathway, leading to hepatic steatosis and hepatic stellate cells will activate by generating pro-inflammatory and pro-fibrotic cytokine secretion. (6) TNF induces palmitoylation of RIPK1, which is dependent on ubiquitination of the RIPK1-K63 linkage in complex I. This promotes trans-activation of RIPK1 and promotes cell death, ultimately leading to liver injury. ZDHHC5 can be amplified by fatty acids in MASH livers. MASH, metabolic dysfunction-associated steatohepatitis; ZDHHC, zinc finger DHHC domain-containing protein; APT, acyl protein thioesterase; PPT, palmitoyl-protein thioesterase; FA, fatty acid; FAO, fatty acid oxidation; ACSL1, long-chain acyl-CoA synthetase 1; AMPK, monophosphate-activated protein kinase; JNK, c-Jun NH(2)-terminal kinase; KLF10, Krüppel-like factors 10; AEG-1, astrocyte-elevated gene-1; IRHOM2, inactive rhomboid protein 2; TRIM31, tripartite motif containing 31; 4-HNE, 4-hydroxynonenal; RIPK1, receptor-interacting serine/threonine protein kinase 1; ER, endoplasmic reticulum.

CD36 palmitoylation is important in lipid metabolism disorders, mitochondrial dysfunction, and inflammatory activation in MASH (**Table 3** **) (** **Figure 5**). Zhao et al. demonstrated that elevated levels of CD36 palmitoylation in MASH patients and mouse models promote plasma membrane localization, enhancing fatty acid uptake and formation of CD36/Fyn/Lyn complexes. It exacerbates hepatic steatosis with inflammation and fibrosis. Three HepG2 cell models were constructed to demonstrate that inhibition of CD36 palmitoylation could simultaneously activate the AMPK pathway to improve lipid metabolism and inhibit the JNK pathway to reduce inflammation (205). Moreover, Zeng et al. found that the inhibition of CD36 palmitoylation induces its mitochondrial membrane translocation, accelerates the conversion of low-fat fatty acids to acyl-coenzyme A through interaction with ACSL1, enhances mitochondrial fatty acid oxidation (FAO) in the liver, reduces ROS accumulation, and ultimately alleviates MAFLD lipid deposition and inflammatory responses in HFD-fed mice (153). Remarkably, multiple transcription factors in hepatocytes can promote or alleviate MASH by regulating the levels of key enzymes of palmitoylation. KLF10 (Krüuppel-like factor 10) promotes the progression of MASLD to MASH through transcriptional upregulation of ZDHHC7 to promote palmitoylation and plasma membrane localization of CD36 (154). However, one of the mechanisms by which Atf3 (activating transcription factor 3) alleviates MASH is by increasing the expression of ZDHHC4/5 and ABHD17A to increase CD36 palmitoylation levels in liver macrophages and enhance fatty acid uptake and FAO (155). These findings suggest that palmitoylation modification is able to regulate the subcellular localization and biological functions of CD36, and that targeting hepatic CD36 palmitoylation modification may be a novel strategy for the treatment of MASH. Significantly, the enzymes regulating CD36 palmitoylation are distinctly cell-type specific, suggesting that the development of therapeutic regimens capable of precisely targeting CD36 palmitoylation-modifying enzymes in specific cells will be a highly promising direction for future research.

Excessive fatty acid accumulation can affect multiple ZDHHC enzymes, regulating the level of associated protein palmitoylation and further activating inflammation-related pathways (**Figure 5**). Abnormal accumulation of palmitic acid can increase Akt and RIPK1 palmitoylation by increasing ZDHHC17/24 and ZDHHC5, respectively, thereby exacerbating hepatocyte death, liver injury, and fibrosis formation in MASH mice. However, sustained Akt activation promotes abnormal hepatocyte proliferation and inhibits apoptosis, driving the transformation of MASH to HCC (hepatocellular carcinoma) (156, 157). Tan et al. found that caffeine inhibits AKT/mTORC1 signaling by blocking FASN-dependent MyD88 palmitoylation, which ameliorates hepatic steatosis and inflammatory injury in MASH mice *in vivo* (206). Hepatocyte-specific knockdown of *Zdhhc3* significantly ameliorated high-fat-hypercholesterolemic diet-induced pathological changes in the mouse liver, including attenuation of lipid deposition, reduction of inflammatory response, and reduction of collagen deposition. In terms of molecular mechanisms, ZDHHC3 loss of function resulted in reduced palmitoylation modification of inactive rhodopsin 2 (IRHOM2), which in turn promoted its degradation via the ubiquitin-proteasome pathway and ultimately inhibited the activation of the downstream MAP3K7-JNK signaling pathway (158). Notably, a chronic high-fat diet positively regulates ZDHHC3 expression, exacerbating hepatic lipid deposition, a process that in turn further promotes ZDHHC3 expression, creating a self-reinforcing vicious cycle. On the therapeutic side, the palmitoylation inhibitor 2-bromopalmitate (2-BP) was shown to improve MASH symptoms, which provides an experimental rationale for the development of therapeutic strategies targeting palmitoylation modifications (158).

Enhanced macrophage STING palmitoylation in the liver activates downstream STING signaling and promotes pro-inflammatory and pro-fibrotic cytokine secretion, leading to hepatic steatosis and hepatic stellate cell activation (159). Komaniecki et al. demonstrated that palmitoylation of AEG-1 Cys75 was able to inhibit signals such as inflammation, lipid accumulation, and motility and their upstream pathways in hepatocytes, suggesting that aberrant AEG-1 palmitoylation may be present in MASH, and that the specific mechanism needs to be further investigated (160). Saverino et al. reported that AEG-1-C75S leads to dysregulation of specific differential gene expression in the hepatic periportal, mid-lobular, and pericentral regions, impairs hepatic metabolic homeostasis, and promotes MASH progression (161). Furthermore, several protein substrate mechanisms involved in lipid synthesis, ROS generation, and enzyme activity regulation are compromised in ZDHHC13-deficient mice, leading to hepatocyte lipid metabolism disorders and mitochondrial dysfunction. These findings imply that ZDHHC13 or its substrate palmitoylation may be a viable therapeutic target for MASH (207).

### 4.4 Sepsis

Sepsis is an infection-induced systemic inflammatory response disease (208). Patients with sepsis are at high risk for infectious shock, disseminated intravascular coagulation, and multiple organ dysfunction syndrome (209). The high mortality rate of sepsis is caused by many serious complications, including acute kidney injury, liver damage, cardiomyopathy, and thrombosis and coagulopathy (210). The pathogenesis of sepsis is complex, and further research is needed to provide new ideas for diagnosing and treating sepsis.

Recent studies have revealed that protein palmitoylation modifications play a central role in the pathological process of sepsis by dynamically regulating inflammatory signaling and organelle function (**Table 3**). In septic mice, decreased GSDMD palmitoylation reduces organ damage and enhances survival by preventing IL-1β release and macrophage pyroptosis (135). NU6300 blocks GSDMD palmitoylation and activation by binding to the palmitoylation site of GSDMD, thereby exerting a therapeutic effect on sepsis (147). Palmitoylation may be a key factor influencing protein sorting into extracellular vesicles (82). In a sepsis model, *Zdhhc21^dep/dep^* mice exhibited reduced levels of palmitoylation, leading to a decrease in the complement component of EVs, which in turn attenuated neutrophil activation and their lung infiltration, ultimately significantly improving survival (162). FASN inhibitor C75 treatment increases survival in septic mice by improving neutrophil chemotaxis through inhibition of MYD88 palmitoylation (62).

Dynamic homeostasis of the palmitoylation cycle plays a key role in sepsis-related complications. Abnormal activation of STING in platelets is a key mechanism in the development of sepsis pathology. Elevated plasma and platelet cGAMP levels in cecal ligation and puncture (CLP)-operated mice activate STING palmitoylation by stimulating its interaction with STXBP2, triggering SNARE complex formation, granule secretion, and platelet activation. It exacerbates sepsis-induced intravascular thrombosis and neutrophil extracellular trap formation (NETosis) in mice (163). Notably, FASN inhibitors ameliorate sepsis-induced liver injury by suppressing STING palmitoylation levels in macrophages by inhibiting palmitate production and promoting propadiene cofactor accumulation (211). Reduced CD36 palmitoylation in LPS-stimulated CD36OE HepG2 cells promotes endocytosis and translocation of CD36 to the lysosomal membrane and autophagic SNARE proteasomal degradation, leading to impaired autophagosome-lysosome fusion and septic liver injury (164). LPS stimulation of H9c2 cells experimentally revealed that in septic cardiomyopathy, the level of palmitoylation of NLRP3 and the interaction of NLRP3 with ZDHHC12 were inhibited, leading to overactivation of NLRP3 inflammatory vesicles (165). Inadequate perfusion/hypoxia of renal tissue is an essential factor in renal insufficiency during septic injury (212). Defective ZDHHC21 function protects renal function and structure during sepsis injury by attenuating the reduction of renal blood flow, renal perfusion, and renal oxygen saturation. Its mechanism involves inhibiting palmitoylation of α1-adrenergic receptor (α1AR), which activates downstream effector molecules and mediates phenylephrine-induced renal artery vasoconstriction (166).

### 4.5 Alzheimer’s disease

Alzheimer’s disease (AD) is one of the most common neurodegenerative disorders induced by chronic neuroinflammation with increased activation of microglia and astrocytes, leading to cognitive impairment and dementia (213, 214). Neuropathological characteristics of the AD brain include the accumulation of amyloid-β plaques (Aβ) and tau-tangle phosphorylation (215). The accumulation and deposition of Aβ in the brain appears to occur early in Alzheimer’s disease and triggers other processes that lead to dementia, including tau phosphorylation, inflammation, synaptic dysfunction, and neuronal loss (213, 216).

Palmitoylation drives protein homeostatic imbalance, abnormal Aβ metabolism, and synaptic dysfunction in the pathological process of AD through multiple mechanisms (**Table 2**). Guo et al. demonstrated that ZDHHC5-mediated palmitoylation of beclin 1 serves as a core mechanism for maintaining neuronal autophagy, and its deficiency in AD murine models impairs autophagic function, exacerbating proteotoxic deposition, thereby leading to neurodegeneration, long-term potentiation (LTP) deficits, and memory deficits (167). Genetic factors are equally important pathological drivers of AD (217). A novel heterozygous missense mutation (ZFHHC21 p.T209S), located in exon 6, was identified in the Han Chinese AD family line. The ZDHHC21T209S/T209S mouse model confirms that the mutation significantly increases neuronal excitotoxicity susceptibility and triggers synaptic dysfunction by enhancing FYN palmitoylation, leading to NMDAR2B overactivation and dysfunction and neuronal loss, ultimately resulting in cognitive deficits (168). The palmitoylation inhibitor 2-BP corrected the abnormal palmitoylation level of FYN and repaired the synaptic function, but its therapeutic effects in animals need to be further investigated.

At the same time, palmitoylation is deeply involved in disease progression by dynamically regulating the Aβ production and clearance pathway. β-site amyloid precursor protein-cleaving enzyme 1 (BACE1) and γ-secretase are key rate-limiting enzymes in the cleavage of amyloid APP to produce Aβ (218, 219). Palmitoylation of both BACE1 and γ-secretase is positively correlated with Aβ burden in the brain (170, 220). In an AD amyloid mouse model lacking BACE1 palmitoylation, reduced BACE1-containing localization within dystrophic neuronal synapses around amyloid plaques led to a significant reduction in local amyloid load, which alleviated cognitive dysfunction in mice (169). ZDHHC7 may be involved in AD pathogenesis by regulating BACE1 palmitoylation, and its expression is upregulated in the hippocampus of 3×TG-AD mice and AD patients. Targeting ZDHHC7 may be a potential therapeutic strategy for AD, but brain region-specific palmitoylase expression differences need to be taken into account, emphasizing the importance of precisely targeted therapy (221). APP palmitoylation promotes its target lipid rafts as BACE1-preferred substrates and increases Aβ production (171). Bhattacharyya et al. demonstrated that palAPP was specifically enriched in the MAM region of human neurons and the mouse brain. *In vitro* experiments confirmed that MAM-mediated membrane transport of palAPP significantly enhanced β-secretase cleavage and Aβ production. However, the mechanism of APP palmitoylation in AD still needs to be deeply elucidated (222). ZDHHC12, a palmitoyltransferase interacting with APP, inhibits Aβ production and pathological deposition by blocking APP transport and enhancing non-amyloidogenic α-cleavage, suggesting that ZDHHC12 may be involved in the early pathogenesis of AD (223).

Furthermore, increased TRPV2 palmitoylation in the brains of AD mice inhibits Aβ phagocytosis in microglia, accompanied by a decrease in their phosphorylation levels, suggesting that the tyrosine phosphorylation cycle and the cysteine palmitoylation cycle have opposing effects on transient receptor potential vanilloid 2 (TRPV2) channel-mediated Aβ phagocytosis in microglia (172). *In vivo* and *in vitro* Se supplementation experiments confirmed that increased levels of SELENOK in the brain attenuated the deterioration of AD by promoting CD36 palmitoylation and enhancing microglia Aβ phagocytosis through interaction with ZDHHC6 (173). Palmitoylated prolactin-releasing peptide (palm11-PrRP31) reduces Aβ plaques and attenuates neuroinflammation in APP/PS1 mouse model (174).

The molecular mechanism of synaptic dysfunction, an early feature of AD, is closely related to palmitoylation modification. Dore et al. found that treatment of WT mice with the exogenous depalmitoylation inhibitor Palm B was able to significantly increase synaptic postsynaptic density protein 95(PSD-95) levels by inhibiting PSD-95 depalmitoylation, which could reverse Aβ-induced synaptic inhibition, but the same manipulation was not effective in PSD-95 knockout (KO) mice. Thus, selective blockade of PSD-95 depalmitoylation may serve as a viable therapeutic option for the development of AD treatments (175). Cerebroside (CEGI) reduces Aβ deposition and ameliorates cognitive dysfunction in APPswe/PS1dE9 AD model mice by decreasing the level of PSD-95 palmitoylation while up-regulating the expression of synaptic proteins, such as NR2B, SYT1, and PSD-95, in the frontal cortex (176). ZDHHC22 is an immunomodulatory target for the potential diagnosis of Alzheimer’s disease and may affect neuroinflammation and immune cell (microglia and T cells) activity, synaptic dysfunction (224).

### 4.6 Parkinson’s disease

Parkinson’s disease (PD) is a rapidly progressive neurodegenerative disorder for which effective disease-modifying therapies are lacking. Studies on its pathogenesis are mainly based on genetic and molecular pathological evidence, with abnormal aggregation and deposition of α-synuclein (αS) as the central pathological feature (225).

Although αS itself cannot be modified by palmitoylation because it lacks cysteine residues, the palmitoylation status of other regulatory proteins can significantly affect αS inclusion body formation (**Table 3**). Defects in αS-dependent vesicular transport are an important pathogenetic mechanism in PD. Palmitoylated Synaptotagmin-11(Syt11) promotes aberrant αS aggregation by decreasing αS tetramerization and increasing its aggregation-prone monomer, suggesting that the two functionally related vesicular transport proteins may synergistically regulate αS homeostasis through palmitoylation in mouse primary neurons and in cells of origin from familial Parkinson’s disease patients (177). PD cell model experiments confirmed that pathological αS (e.g., 3K mutants or E46K) impairs vesicle trafficking and forms inclusion bodies by accelerating APT1-mediated depalmitoylation of microtubule-associated-protein-6 (MAP6) and impairing its ability to bind to vesicles. Reduced MAP6 palmitoylation was similarly observed in neurons of patients with familial PD, and inhibition of APT1 reduced αS inclusion bodies and alleviated neurotoxicity. Targeting the APT1-MAP6 axis may be a potential therapeutic strategy (178). The depalmitoylation inhibitor ML348 was able to ameliorate synaptic dysfunction and cognitive-motor deficits in αS transgenic mice by inhibiting the palmitoylation of estrogen receptor α (ERα). However, since ML348 inhibits other substrates of APT1 as well, its action is not ERα-specific, suggesting that the development of specific drugs targeting palmitoylation of ERα still requires further studies (179). Cervilla-Martínez et al. found that multiple abnormalities of palmitoylated proteins were present in PD patients, which were strongly associated with mitochondrial dysfunction, oxidative stress, and inflammation (226). Glial cell-derived neurotrophic factor (GDNF) effectively protects nigral dopaminergic neurons from 6-hydroxydopamine (6-OHDA) damage by promoting palmitoylation modification of neural cell adhesion molecules (NCAMs), which significantly enhances their localization in lipid raft microregions. This protective effect was characterized by a significant increase in cell viability, a decrease in apoptosis, and an inhibition of caspase-3 activation, while 2-BP pretreatment attenuated GDNF-induced redistribution of NCAM to lipid rafts (227).

### 4.7 Diabetes mellitus

Diabetes mellitus (DM) is a chronic metabolic disorder characterized by hyperglycemia, and its increasing global prevalence has become a major public health challenge (226, 228). Based on differences in pathogenesis, DM is mainly classified into type 1 diabetes mellitus (T1DM) and type 2 diabetes mellitus (T2DM), of which T1DM is insulin-dependent. T2DM is the most common type of diabetes mellitus, accounting for 90–95 percent of cases, and is characterized by insulin resistance and/or β-cell dysfunction (229, 230). Prolonged hyperglycemic state can lead to multi-organ damage such as diabetic foot, nephropathy, retinopathy, and cardiovascular disease (231). Significantly, the inflammatory response is associated with the development and progression of DM and its complications (224).

Growing evidence that palmitoylation plays a key regulatory role in diabetes and its complications (**Table 3**). ZDHHC7 significantly facilitates the translocation of insulin-dependent translocation of glucose transporter 4 (Glut4) to the plasma membrane by catalyzing its palmitoylation modification, a process that is essential for maintaining glucose homeostasis in the body. Notably, *Zdhhc7* KO mice exhibited significant hyperglycemia and glucose intolerance phenotypes, a finding that not only confirms the central role of the ZDHHC7-Glut4 pathway in glucose metabolism regulation but also suggests that the defective palmitoylation of Glut4 may be an important link in the pathogenesis of diabetic hyperglycemia (232). The three palmitoylation-related genes, MNDA, FCGR3B, and AQP9, may influence insulin-related signaling and, thus, the progression of gestational diabetes mellitus (233). Decreased endothelial APT1 enzyme activity in diabetic patients suggests its involvement in diabetes progression; APT1 deficiency has been shown by both *in vivo* and *in vitro* experiments to lead to abnormal insulin secretion and β-cell failure, and defective palmitoylation of Scamp1 effectively reverses this pathological phenotype. Together, these findings reveal a critical role for the APT1-Scamp1 palmitoylation axis in the maintenance of β-cell function and glucose homeostasis (234).

Peroxiredoxin-6 (PRDX6) and PEX11B palmitoylation can affect diabetic neuropathy (181, 182). The level of palmitoylation modification of the SMPDL3B protein was significantly upregulated in retinal tissues of diabetic mice, which ameliorated retinal vascular endothelial dysfunction by enhancing its protein stability and inhibiting the activation of NF-κB/NLRP3 inflammatory pathway. This finding provides a new target for the treatment of diabetic retinopathy (DR), but the changes in the expression of ZDHHC5, a key enzyme that regulates the palmitoylation of SMPDL3B, in patients with DR need to be further verified (183). *In vitro*, Ras palmitoylation promotes stress kinase activation through the Tiam1-Rac1-Nox2 signaling module, leading to mitochondrial dysfunction and endothelial cell apoptosis that drive the progression of diabetic retinopathy, whereas 2-BP is able to inhibit high-glucose-induced p38 MAPK activation and mitochondrial DNA damage, and targeted intervention of these pathways may provide a new strategy for the early control and prevention of diabetic vision loss (235). Hyperglycemia in diabetes mellitus reduces APT1 activity, causing impaired palmitoylation of proteins like R-Ras in endothelial cells, triggering abnormal fibronectin metabolism and matrix build, and promoting renal injury and subendothelial fibronectin-triggered vascular inflammation (184). Reduced palmitoylation of endothelial nitric oxide synthase (eNOS) in insulin-deficient or insulin-resistant diabetic mice induces endothelial dysfunction manifested as inflammation and impaired angiogenesis (180). Increased CD36 palmitoylation in TGR5^ΔCM^ diabetic mice promotes fatty acid uptake and lipid accumulation, leading to cardiac hypertrophy and dysfunction. Knockdown of ZDHHC4 in cardiomyocytes reversed the increase in CD36 palmitoylation induced by TGR5 deletion, suggesting that the TGR5-ZDHHC4 pathway is a key target for intervening in lipid metabolism in diabetic cardiomyopathy (185). The pro-inflammatory phenotype of macrophages from diabetic foot ulcer patients is primarily caused by increased binding of accumulated phenylpyruvic acid to PPT1, which inhibits depalmitoylation activity and promotes NLRP3 palmitoylation and stability, which in turn activates the NLRP3 inflammasome and releases inflammatory factors (77).

## 5 Conclusion and perspective

As a dynamically reversible post-translational modification of proteins, S-palmitoylation serves as a central hub for mediating downstream signaling pathways by precisely regulating the subcellular localization, conformation, stability, and molecular interaction networks of key proteins in inflammatory signaling pathways. In a variety of inflammatory diseases, including IBD, AIDs, and sepsis, aberrant palmitoylation modifications drive cascading amplification of inflammatory signals by disrupting protein membrane localization (CD36), interfering with nucleocytoplasmic shuttling (STAT3), or weakening protein complex stability (NLRP3 inflammasome). Remarkably, the regulation of protein transport and localization by palmitoylation dominates disease mechanisms. Different palmitoylation modifications exist at different stages of the inflammatory pathway, and together, they maintain the inflammatory homeostasis of the organism. A variety of compounds can promote or inhibit the development of inflammatory diseases by affecting palmitoylation modifications and their associated enzyme activities. Furthermore, palmitate synthesis and uptake have important roles in palmitoylation modifications, such as FASN affects the palmitoylation level of proteins by regulating the intracellular palmitate pool, whereas palmitoylation of CD36 is able to regulate its membrane localization, which in turn affects the uptake of free fatty acids, and this regulatory mechanism provides a new interventional perspective for metabolic and inflammatory diseases such as non-alcoholic hepatitis. Further studies have shown that palmitoylation-related enzymes have an important role in the pathological process of disease. However, there may be palmitoylated substrates that have not yet been discovered or whose palmitoylase activity has no effect on disease. The other functions of these enzymes are of great value to study. Interestingly, the same protein has different palmitoylating enzymes in different tissues and cells, and their functions differ. An in-depth study of these functional differences and their specific enzymes will provide new ideas and directions for the study of inflammation-related diseases.

The research on palmitoylation has advanced dramatically in recent years thanks to the development of chemical tools to study palmitoylation, such as radiolabeling, acyl-biotin exchange (ABE), and acyl resin assisted capture (acyl-Rac), acyl-PEG exchange (APE), etc., and the Click reaction (236) (**Table 4**). The use of these tools and proteomic approaches allows the analysis of S-palmitoylation in different cell types and contributes to the development of computer prediction of palmitoylation sites in proteins (245). Interestingly, multiple regulatory mechanisms exist for palmitoyltransferase and depalmitoyltransferase activity. ZDHHC is known to be associated with accessory proteins (GOLGA7 (also known as GCP16), huntingtin, and selenoprotein K) that regulate their stability, activity, and transport (246). A variety of post-translational modifications, such as phosphorylation, ubiquitination, methylation, and palmitoylation, are present in ZDHHC, and they have important roles in regulating ZDHHC enzyme stability, localization, and activity. Few mechanisms are known to regulate APT activity. Palmitoylation and ubiquitination control the size of the APT2 pool and its localization and activity, and APT1 also undergoes palmitoylation (36).

**Table 4:** Palmitoylation detection methods: technical principles, advantages, and limitations.

Detection method	Technical principle	Advantages	Limitations
ABE (acyl-biotin exchange) assay	1. Sequestration of free cysteine 2. Hydroxylamine cleavage of thioester bonds to expose palmitoylated cysteines 3. Biotin labeling enrichment	1. Proteome-wide S-acylation status can be analyzed 2. Compatible with downstream analysis by mass spectrometry 3. High sensitivity (low-abundance proteins can be detected)	1. Inability to distinguish between specific S-acylation types (e.g., palmitoylated vs. cardamoylated) 2. Modification stoichiometry cannot be determined 3. Hydroxylamine may cause non-specific cleavage 4. The high background noise caused by the capture of non-S-palmitoylated proteins
Acyl-RAC (acyl resin-assisted capture) assay	1. Sequestration of free cysteine 2. Hydroxylamine cleaves the thioester bond 3. direct covalent binding of exposed sulfhydryl groups to thiol resins	1. High-throughput screening of S-acylated proteins (especially for micro samples) 2. Simplified procedure with low sample loss 3. Low background signal	1. Limited resin binding capacity 2. Need to optimize batch consistency
APE(Acyl-PEG exchange assay)	1. Sequestration of free cysteine (NEM) 2. hydroxylamine (NH_2_OH) cleavage of thioester bonds to release palmitoylated cysteine 3. PEG-maleimide (mPEG-Mal) labeling of exposed sulfhydryl groups 4. SDS-PAGE/WB detection of molecular weight shift	1. Direct quantification of endogenous modification levels (stoichiometric ratios) 2. No enrichment step required 3. High sensitivity (can detect endogenous proteins)	1. Cannot differentiate between specific lipoyl chain types (e.g., C16 vs C18) 2. Need to optimize PEGylation conditions (avoid NH_2_OH interference)
Metabolic labelling	1. Cellular uptake of radiolabeled or click chemistry compatible lipids 2. Detection of modified proteins by gel electrophoresis (for radiolabeling) or click chemistry methods	1. Enables monitoring of modification turnover (by using different labeling times for radiolabeled or clickable lipids) 2. Facilitates proteomic analysis 3. Provides information on lipid type 4. Distinguish fatty acids linked to proteins from other types of cysteine thioesters (e.g., ubiquitin) 5. Study changes in palmitoylation dynamics (e.g., pulse tracking experiments)	1. Synthetic lipid analogs may not fully mimic the natural substrate 2. Detect modifications occurring during labeling only 3. Requires hydroxylamine sensitivity to verify S-palmitoylation
TIRFM Imaging (Total Internal Reflection Fluorescence Microscopy)	1. Selective excitation of cell membrane surface fluorescence signals using evanescent waves generated by total internal reflection (~100–200 nm depth) 2. Comparison of membrane localization differences between wild-type (WT) and palmitoylation-deficient mutants (Cys→Ser)	1. Ultra-high signal-to-noise ratio (detects only proximal membrane signals) 2. Live cell compatibility 3. millisecond time resolution 4. Real-time observation of membrane microcellular distribution of palmitoylated proteins	1. Membrane surface protein observation only 2. Dependent on fluorescent tags (e.g. GFP fusion proteins)
Palmitoylation site prediction software (CSS-Palm 2.0/CKSAAP-Palm)	Prediction of cysteine palmitoylation probability based on machine learning algorithms (sequence features, amino acid frequencies)	1. Free online tools 2. High-throughput screening	1. High false positive rate 2. Cannot reflect dynamic modifications
Acyl-cLIP (acylation-coupled lipophilic induction of polarization)	1. hydrophobic lipid modifications in the lipidation reaction cause the fluorescently labeled peptide to bind to a lipid-binding molecule (e.g., descaler micelles or BSA) 2. After binding, molecular motion is slowed down, and the fluorescence polarization (fatty acid) signal is enhanced. 3. Real-time monitoring of fatty acid changes to quantify enzyme activity	1. Applicable to a variety of lipid modifications (S/N-palmitoylation, N-cardamoylation, farnesylation, etc.) 2. Real-time monitoring 3. Non-radioactive: using natural lipid substrates to avoid isotope risk 4. High throughput compatible	1. Peptide design limitation 2. Lipid carrier required 3. Not suitable for *in situ* detection of membrane proteins

Although targeting palmitoylation modifications offers new opportunities for the treatment of inflammatory diseases, their clinical translation still faces several difficulties (**Table 5**). To date, no therapeutic agents have been developed to modulate specific ZDHHC enzymes. Unlike the kinases for which multiple inhibitors have been developed and tested in clinical trials, the most common palmitoylation inhibitor, 2-BP, cannot be used clinically because of its significant off-target activity and toxicity, including inhibition of mitochondrial fatty acid oxidation (247, 248). The broad-spectrum thioesterase inhibitor palmostatin B also has off-target effects and poor drug specificity, leading to a lack of stability, thus limiting its clinical use (249, 250). ZDHHC family members and depalmitoylated enzymes are generally multi-substrate specific, and there are still substantial gaps in the knowledge of their substrate profiles and tissue-specific regulation patterns, which further complicates targeted palmitoylation therapy. For future exploration, an immediate goal is to develop selective ZDHHC inhibitors as well as novel pharmacological APT protein inhibitors. Given that all ZDHHC family members contain highly conserved DHHC catalytic structural domains, systematic off-target effect assessment must be performed in the design of selective inhibitors, a critical step that will effectively address the problem of non-specific inhibition caused by structural homology. Through this rigorous validation approach, the interference of inhibitors with other cellular signaling pathways can be minimized, thus significantly improving the targeting of drug design. The implementation of this strategy is not only expected to lead to the development of more selective ZDHHC inhibitors, but also to provide new therapeutic options to alleviate the associated disease burden. Moreover, considering that ZDHHC as a membrane-bound protein is mainly localized in organelle membranes such as the endoplasmic reticulum and Golgi apparatus, the design of inhibitors has to take into account the balance between the efficiency of transmembrane delivery and the physicochemical properties of the drug, in particular the need to optimize the molecule’s lipophilicity and water solubility parameters. More importantly, since the same ZDHHC enzyme or acyltransfer protein (APT) may regulate different substrate protein palmitoylation processes in different tissues and cell types, the development of specific drugs that can precisely target disease-related tissues or cells is of critical therapeutic importance.

**Table 5:** Therapeutic agents targeting protein palmitoylation.

Drug	Target protein	Function	Diseases	References
2’-fucosyllactose	STAT3	Restoration of the intestinal mucosal barrier by inhibiting STAT3 phosphorylation and palmitoylation	Inflammatory bowel disease	(143)
Metformin	Akt	Inhibition of Akt palmitoylation by reduction of FASN attenuates macrophage inflammation	Inflammatory bowel disease	(146)
ABD957	ABHD17	Inhibition of ABHD17 to increase palmitoylation of NOD2 enhances NOD2 recognition of bacteria	Inflammatory bowel disease	(149)
NU6300	GSDMD	Inhibition of palmitoylation and cleavage of GSDMD by covalent binding to Cys191 of GSDMD reduces pyroptosis-mediated inflammation	Inflammatory bowel disease	(147)
HDSF	PPT1	Inhibition of IFNα secretion by TLR9 depalmitoylated plasmacytoid dendritic cells (pDC) and TNF secretion by macrophages	Systemic lupus erythematosus	(60)
CP113,818	ACAT1	Reduces the generation of palmitoyl-CoA production by inhibiting ACAT1 activity, thereby reducing the level of palmitoylation of APP and ultimately Aβ production	Alzheimer’s disease	(171)
2-BP		Blocking ZDHHC-mediated S-palmitoylation by direct and irreversible blockade of acyl intermediate formation	Alzheimer’s disease, metabolic dysfunction-associated steatohepatitis, and inflammatory bowel disease	(158, 221, 237)
palmostatin B	APT1, APT2	Increased SQSTM1/p62 palmitoylation restores autophagy	Huntington’s disease	(238)
ML348	APT1	Inhibition of beta-catenin depalmitoylation prevents ischemia/reperfusion injury (IRI)-induced renal fibrosis.	Ischemia/reperfusion injury (IRI)	(239)
ML349	APT2	1. Promotes MAVS palmitoylation through inhibition of APT2, which in turn promotes antiviral signaling. 2. Promoted GPX4 palmitoylation by inhibiting APT2 and reduced ischemia-reperfusion-induced liver injury.	Viral infection	(196, 240)
Caffeine	MYD88	Reduction of MYD88 palmitoylation by inhibition of *de novo* fat synthesis ameliorates hepatic steatosis and inflammatory injury	Metabolic dysfunction-associated steatohepatitis	(206)
Vaccarin	ZDHHC12, NLRP3	Promoted NLRP3 palmitoylation to inhibit inflammatory vesicle activation and attenuate septic myocardial injury	Septic cardiomyopathy	(165)
α-keto-epoxy	N-Ras, H-Ras	Exhibited higher efficacy in inhibiting palmitoylation of N-Ras and H-Ras proteins without affecting fatty acid synthesis	Cancer	(241)
tunicamycin	–	Direct blockade of palmitate transfer to proteins *in vitro*	–	(242)
curcumin	ZDHHC3	Blocked self-acylation of ZDHHC3, which is responsible for integrin (ITGβ4) palmitoylation	–	(243)
Disulfiram	GSDMD	Inhibition of Cys192 Palmitoylation at the Terminal End of GSDMD-N and Reduction of Cardiomyocyte Focal Death and Injury in AMI Mice	Myocardial infarction	(244)
selenoprotein K (SELENOK)	ZDHHC6	Promotion of CD36 palmitoylation via ZDHHC6 enhances CD36 expression on microglia membranes and ultimately significantly enhances Aβ phagocytosis	Alzheimer’s disease	(173)
Cattle Encephalon Glycoside, Ignotin	PSD-95	Enhancement of synaptic protein expression by increasing PSD-95 palmitoylation	Alzheimer’s disease	(176)
Fenofibrate		Reduced PA-induced PEX11B palmitoylation modification to treat diabetic neuropathy	Diabetic neuropathy	(182)
C-170, H-151, nitrofatty acids (NO2-FAs), BPK-21, and 4-octyl itaconate	STING	Inhibited STING palmitoylation by covalently binding to STING Cys88/91	Antiviral immune responses, autoimmune inflammation	(103–105)

STAT3, signal transducer and activator of transcription 3; Akt, Ak strain transforming; FASN, fatty acid synthase; GSDMD, gasdermin-D; ACAT1, acetyl-coA acetyltransferase 1; APP, amyloid precursor protein; Aβ, myloid-β plaques; MAVS, mitochondrial antiviral-signaling protein; GPX4, glutathione peroxidase 4; MYD88, myeloid differentiation primary response protein 88; PSD-95, postsynaptic density protein 95.

In addition to treating diseases by modulating palmitoylation-related enzymes, therapeutic agents can target specific palmitoylated cysteine residues, such as C176/178, H-151, NO2-FAs or nitrofuran molecules, which can block STING palmitoylation modification by covalently modifying the Cys91 residue of STING, inhibiting type I interferon signaling, and providing a new therapeutic target for STING-associated diseases by blocking STING palmitoylation and inhibiting type I interferon signaling (103, 251). Palmitoylation site-specific modulation has significant potential as a novel therapeutic strategy for inflammatory diseases, which centers on the development of small molecule compounds capable of targeting specific protein palmitoylation modifications. To achieve this goal, there is an urgent need to establish efficient and reliable high-throughput palmitoylation assay platforms for compound screening. Existing studies have shown that some ZDHHC enzyme family members exhibit specific expression patterns in different tissues (252), however, the molecular mechanisms that regulate this tissue-specific enrichment remain to be elucidated in depth. Notably, from the perspective of therapeutic strategies, precise modulation targeting palmitoylation modification of key functional proteins in specific tissues may exhibit more significant clinical translational value than relying solely on the tissue distribution profile of ZDHHC enzymes.

Overall, palmitoylation modifications are closely associated with inflammatory diseases. Although existing studies have tentatively confirmed the prevalence of dysregulated palmitoylation levels in inflammatory diseases, their dynamic regulatory mechanisms and pathological roles are limited, and the therapeutic agents for palmitoylation are still in their infancy, so more in-depth studies are needed to explore the clinical significance of palmitoylation modification in inflammatory diseases.

## Glossary

ABE: Acyl-biotin exchange
ABHD: α/β
Acyl-Rac: acyl resin assisted capture
AD: Alzheimer’s disease
AEG-1: Astrocyte elevated gene-1
AIDs: Autoimmune diseases
APE: Acyl-PEG exchange
APP: Amyloid precursor protein
APT: Acyl protein thioesterase
ASC: Apoptosis-associated speck-like protein containing a CARD
Atf3: Activating transcription factor 3
Aβ: Amyloid-β
BACE1: β-site amyloid precursor protein-cleaving enzyme 1
CEGI: Cattle encephalon glycoside
cGAMP: Cyclic GMP-AMP
cGAS: Cyclic GMP-AMP synthase
CMA: Chaperone-mediated autophagy
CTD: C-terminal containment domain
DAMP: Damage-associated molecular pattern
DM: Diabetes mellitus
dTGN: Dispersed TGN
eNOS: Endothelial nitric oxide synthase
FAO: Fatty acid oxidation
FASN: Fatty acid synthase
Glut4: Glucose transporter 4
GRK6: protein-coupled receptor kinases 6
GSDMD: Gasdermin-D
HCC: Hepatocellular carcinoma
HSC70: Heat shock cognate protein of 70 kDa
IBD: Inflammatory bowel disease
iE-DAP: γ-d-glutamyl-meso-diaminopimelic acid
IKK: IκB kinase
IRAK: Interleukin-1 receptor-associated kinase
IRF3: Interferon regulatory factor 3
IRHOM2: Inactive rhomboid protein 2
KLF10: Krüuppel-like factors 10
LCFAs: Long-chain fatty acids
LPS: Lipopolysaccharide
LRR: Leucine-rich repeat
LTP: Long-term potentiation
MAPK: MAP kinases
MASH: Metabolic dysfunction-associated steatohepatitis
MASLD: Metabolic dysfunction-associated steatotic liver disease
MAVS: Mitochondrial antiviral-signaling protein
MDP: Muramyl dipeptide
MTOC: Microtubule organizing center
MUC2: Mucin 2
MYD88: Myeloid differentiation primary response protein 88
NEK7: NIMA-associated kinase 7
NLR: NOD-like receptor
NOD: Nucleotide-binding oligomerization domain
NR2B: N-methyl-D-aspartic acid receptor subunit 2B
NTD: N-terminal structural domain
PAMP: Pathogen-associated molecular pattern
PAT: Palmitoyl acyltransferase
PD: Parkinson’s disease
pDC: Plasmacytoid dendritic cells
PGN: Peptidoglycan
PPT: Palmitoyl protein thioesterases
PRDX5: Peroxiredoxin 5
PRDX6: Peroxiredoxin-6
PRRs: Pattern recognition receptors
PtdIns4P: Phosphatidylinositol 4-phosphate
PYD: Pyridine structural domain
RIPK2: Receptor-interacting serine/threonine-protein kinase 2
ROS: Reactive oxygen species
SLE: Systemic lupus erythematosus
SMPDL3B: Sphingomyelin phosphodiesterase acid-like 3B
STAT3: Signal transducer and activator of transcription 3
STING: Stimulator of interferon genes
SYT1: Synaptotagmin 1
TAB1: TAK1-binding protein
TAK1: Transforming growth factor-β-activated protein kinase 1
TBK1: TANK-binding kinase 1
TGN: Trans-Golgi network
TLR: Toll-like receptors
TRAF6: Tumor necrosis factor receptor associated factor 6
TRPV2: Transient receptor potential vanilloid 2
ZDHHCs: Zinc finger DHHC-containing proteins
α1AR: α1-adrenergic receptor
αS: α-synuclein.
